# Calcium-Doped High-Voltage Spinel Cathode for Long Cycle Life Lithium-Ion Batteries

**DOI:** 10.1021/acsaem.6c00023

**Published:** 2026-03-17

**Authors:** Jie Xiong, Bingyao Zhou, Kevin Mathew, Emmanuel Kornyo, Guoxin Zhang, Wenquan Lu, Zhi Mei, Qingliu Wu

**Affiliations:** † Department of Chemical and Paper Engineering, 4175 Western Michigan University, 4601 Campus Drive, Kalamazoo, Michigan 49008-5462, United States; ‡ Chemical Science and Engineering Division, Argonne National Laboratory, 9700 South Cass Ave, Lemont, Illinois 60439-4837, United States; § Lumigen Instrument Center, 2954 Wayne State University, 5101 Cass Ave, Detroit, Michigan 48202, United States

**Keywords:** high voltage spinel, doping, calcium, lithium-ion battery, cation ordering

## Abstract

With the promises of low cost, high operating voltage, and excellent rate capability, the high-voltage spinel material with the formula of LiNi_0.5_Mn_1.5_O_4_ (LNMO) has been considered as one of the most promising cathode materials for next-generation lithium-ion batteries (LIBs). However, the adoption of LNMO into practical LIBs is greatly hindered due to its rapid capacity decay associated with its bulk structural instability and interfacial side reactions. To address these issues, we proposed to use the cost-effective calcium (Ca) element as a dopant to stabilize the oxygen framework and surface of the LNMO crystal. The experimental results showed that, with moderate Ca doping, the obtained cathode (Ca 0.05 LNMO) retained a specific capacity of ∼121 mAh/g (∼94.4% capacity retention) after 500 cycles at 0.5 C, compared to ∼73% for the baseline bare sample. Furthermore, the Ca 0.05 LNMO cathode retained ∼84% of its initial capacity, vs the baseline with ∼69%, after 150 cycles at the high temperature of 55 °C. The excellent battery performance of the moderately Ca-doped LNMO cathode is ascribed to its structural and kinetic advantages.

## Graphical abstract

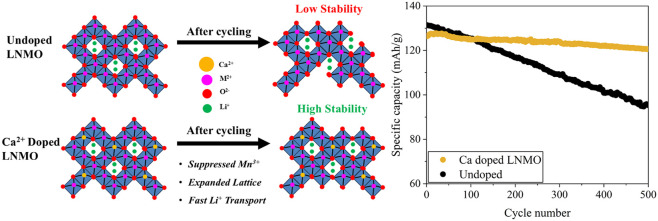

## 1 Introduction

The high-voltage spinel material with the formula of LiNi_0.5_Mn_1.5_O_4_ (LNMO) has the advantages of low cost (Co-free), high operating voltage (∼4.7 V vs Li/Li^+^), and excellent rate capability and has been considered as one of the most promising cathode materials for next-generation lithium-ion batteries (LIBs). However, the adoption of LNMO into practical LIBs is greatly hindered.^1^ One root cause of this limitation is its rapid capacity decay, especially under elevated temperatures, primarily associated with its bulk structural instability, dissolution of transition metals (TMs), and interfacial side reactions between the active material and the liquid electrolyte.^2–4^ Therefore, there is a critical need to improve the structural and surface stabilities of particles to boost the electrochemical performance of LNMO cathode materials.

Various strategies have been developed to address the aforementioned challenges. Surface coating has been widely adopted to suppress parasitic reactions occurring at the electrode–electrolyte interface. It is believed that the presence of an inert surficial coating layer can effectively isolate the active material LNMO particles from the electrolyte, prevent the dissolution of Mn, and mitigate the formation of cathode–electrolyte interface (CEI) films.^5–7^ For instance, the work done by Fang et al. showed that an ultrathin Al_2_O_3_ layer with a thickness of 3–4 nm coated on the surface of LNMO particles can effectively improve the capacity retention of cells to 91% after 200 cycles, compared to only 75% for baseline LNMO without a surficial coating layer.^8^ The conformal Al_2_O_3_ layer acts as a physical barrier that suppresses HF attack, reduces Mn/Ni dissolution, and stabilizes the cathode–electrolyte interphase. Similarly, Kim et al. coated LNMO with a homogeneous layer of nano-LiNbO_3_.^9^ The coated LNMO maintained ∼100 mAh/g even at 10 C at room temperature and ∼90% capacity retention after 100 cycles at 60 °C, demonstrating robust interfacial stabilization at high rates and elevated temperatures. The LiNbO_3_ layer not only passivates the surface but also provides fast Li^+^ transport pathways, thereby reducing the interfacial resistance of LNMO cathodes. Chang et al. investigated the effect of phosphate coating on LNMO.^10^ The AlPO_4_-coated LNMO cycled in the range of 3.5–5.1 V exhibited ∼97.5% capacity retention after 100 cycles, compared to only ∼76% for uncoated LNMO. The improvement was attributed to the effective barrier effect of the AlPO_4_ layer. However, the addition of a surface layer comes at the cost of reduced energy density and does not address the bulk structural instability, which remains one of the main challenges to achieving a long cycle life of LNMO.

An alternative and effective approach to improve the cycling stability of LNMO without significantly sacrificing energy density is elemental doping. Many dopants, such as Mg,^11^ Ti,^12^ Al,^13^ and Cr,^14,15^ have been reported to improve LNMO cycling stability by suppressing Mn^3+^ formation. Liang et al. showed that the Mg^2+^ substitution at both tetrahedral and octahedral sites can suppress Mn dissolution, alleviate Jahn–Teller distortion, and stabilize the bulk structure of the spinel crystal. The optimized Mg 0.1–LNMO retained 94.6% of the initial capacity after 500 cycles and 86.3% after 1500 cycles at 1 C, compared to <70% for undoped LNMO.^11^ Mao et al. reported that Cr–O bonds can reduce the Mn^3+^ concentration, enhance Li^+^ transport across the particles, and thus lower cell polarization. The Cr-doped LiCr_0.1_Ni_0.45_Mn_1.45_O_4_ cathode maintained 94.1% of the initial capacity after 500 cycles at 1 C, compared to ∼82% for undoped LNMO, confirming the stabilizing role of Cr.^14^ Chen et al. investigated the Al doping effect in LNMO. The as-prepared Al-doped LNMO delivered 113.5 mAh/g after 200 cycles at 0.5 C and 25 °C, and also exhibited enhanced high-temperature stability. The improved cycling stability was attributed to an enhanced framework and suppressed TM dissolution/CEI growth.^13^

It is believed that dopants with strong M–O bonds can help stabilize the oxygen framework, reduce lattice oxygen release, thereby suppressing Mn^3+^ formation, and improve the structural integrity of LNMO crystals over the long-term cycling test. Notably, most reported dopants have ionic radii smaller than or close to those of the transition metals that they replace (Ni^2+^ or Mn^4+^), and rare studies on elements with both large ionic radii and strong M–O bonds have been reported as dopants in LNMO cathodes. This may be due to the concern that substituting with large dopants might distort the spinel lattice, hinder Li^+^ diffusion, and undermine the electrochemical performance of LNMO cathodes. However, several studies have shown that when properly incorporated, large dopants can be beneficial to the cycling stability of cathode materials with layered structures. Wang et al. investigated Mg^2+^ and Ca^2+^ doping in Ni-rich layered LiNi_0.8_Co_0.1_Mn_0.1_O_2_ (NCM 811) and found that Ca^2+^ doping (3% mol) improved the cycling stability of the NCM 811 cathode with ∼81% capacity retained after 400 cycles at 1 C, compared to 63.4% for the undoped sample. The improved electrochemical performance is ascribed to the enhanced Li^+^ transportation and reduced surface resistance, although excessive lattice expansion might lead to structural degradation.^16^ DFT analysis further confirmed that larger dopants preferentially occupy Li layers, increase interlayer spacing, and thus facilitate the formation of the diffusion pathways in NCM 811. Similar phenomena have also been observed in LNMO cathodes doped with large ions. The work done by Wei et al. showed that the K^+^/Cl^–^ and K^+^/F^–^ codoped LNMO cathodes exhibited excellent rate capability, delivering 116.1 mAh/g and 93.9 mAh/g at 5 C and 10 C, respectively, and long-term cycling stability with 85.8% capacity retained after 500 cycles at 5 C.^17^ It was found that the excellent electrochemical performance is associated with the expanded lattice, suppressed Mn^3+^ formation, and improved particle uniformity in the selective K^+^/Cl^–^ and K^+^/F^–^ codoped LNMO cathodes. The prior art achievements inspired us to dope elements with large ionic radii and strong oxygen affinity into the LNMO to expand the crystal lattice, stabilize the crystal structure, and eventually boost the rate and cycling capability of cells. Especially, the significant difference in their electronegativities makes the bonds between oxygen (O) and calcium (Ca), strontium (Sr), or chromium (Cr) strong ionic bonds,^18^ which are much stronger than Mn–O, Ni–O, and other M–O bonds (Table S1). In addition, both Ca^2+^ and Sr^2+^ have radii of >1 Å, which are significantly larger than those of Ni^2+^ and Mn^2+^. Therefore, it is rational to expect that an appropriate integration of Ca, Sr, or Cr into the LNMO cathode can significantly boost the cell rate capability and durability through the effectively improved structural and surficial stability, suppressed Mn^3+^ formation, and expanded lattice in LNMO crystals. Despite these advantages, studies on utilizing Ca, Sr, and Cr as dopants in LNMO cathodes have been rarely investigated to improve the electrochemical performance of LNMO cathodes. Furthermore, most prior dopant strategies for LNMO have focused mainly on either bulk structural stabilization or morphology control, whereas a synergetic approach that simultaneously enhances both bulk stability and morphology control has rarely been investigated.

In this work, Sr, Cr, and Ca were preliminarily introduced as dopants in LNMO cathodes, and among these three dopants, the Ca-doped cathode demonstrated the best electrochemical performance in terms of rate capability and cycling stability. Ca was then chosen as the dopant, and the effect of the doping level on the crystal, morphological, and electrochemical properties of LNMO cathodes was further investigated.

## 2 Experimental Section

### 2.1 Chemicals

Manganese nitrate tetrahydrate (Mn­(NO_3_)_2_·4H_2_O, ≥98%, Fisher Chemical), Nickel nitrate hexahydrate (Ni­(NO_3_)_2_·6H_2_O, ≥98%, Fisher Chemical), Calcium nitrate (Ca­(NO_3_)_2_·4H_2_O, ≥99%, Fisher Chemical), Strontium nitrate (Sr­(NO_3_)_2_, 98%, Thermo Scientific Chemicals), Chromium nitrate nonahydrate (Cr­(NO_3_)_3_·9H_2_O, 99%, Thermo Scientific Chemicals), Sodium hydroxide (NaOH, ≥98%, Fisher Chemical), 1-methyl-2-pyrrolidone (NMP, 99%, Acros Organics), Carbon black (C45, Timcal), Polyvinylidene difluoride (PVDF 5130, molecular weight of 1300 kDa, Solvay), Aqueous ammonia solution (NH_3_·H_2_O, 28–30 wt %), Coin cell cases (CR2032, Canrd), and Microporous membrane (39% porosity, Celgard) were used. All of the chemicals used in this study were used without further treatment.

### 2.2 Material Synthesis

Both doped and undoped LiNi_0.5_Mn_1.5_O_4_ (LNMO) cathodes were synthesized in a homemade coprecipitation reactor.

#### 2.2.1 Precursor Synthesis

In a typical synthesis of the LNMO precursor, both metal solutions and base solutions were prepared first. For the metal solution, manganese nitrate (Mn­(NO_3_)_2_·4H_2_O) and nickel nitrate (Ni­(NO_3_)_2_·6H_2_O) at a stoichiometric ratio were dissolved in deionized water. A 4 M NaOH solution with 1 M NH_4_OH in deionized water was used as the base solution. During coprecipitation, 16 mL of metal solution and 20 mL of base solution were simultaneously pumped into a conical flask containing 60 mL of NH_4_OH mother solution using syringe pumps at controlled rates to maintain a stable pH of ∼11. The reaction was carried out under continuous magnetic stirring for an additional 2 h after the completion of solution addition to ensure the complete precipitation of Ni and Mn. Afterward, the obtained precursor was filtered and washed three times with deionized water until the filtrate reached a near-neutral pH (∼7). The collected precursor was then dried at 70 °C overnight in an air oven.

#### 2.2.2 LNMO Synthesis

The dried precursor powder was manually mixed with lithium hydroxide (LiOH) at a molar ratio of Li:(Mn + Ni) = 1.05:1 and ground using an agate mortar and pestle to ensure homogeneous mixing. The mixture was then calcined in a tube furnace under an airflow at a rate of ∼0.1 L/min. A two-step thermal treatment involving initial calcination at 900 °C for 15 h followed by annealing at 700 °C for 8 h was applied during the calcination step. The heating rate was set to 5 °C/min. After being naturally cooled to room temperature, the obtained LNMO samples were collected for further characterization.

For the dopant screening study, the same procedure was used to synthesize the precursors and final LiNi_0.5_Mn_1.4_M_0.1_O_4_ (M = Ca, Sr, Cr) cathodes, except for the metal solution used in the coprecipitation process for precursor synthesis. To prepare metal solutions, Ca­(NO_3_)_2_·4H_2_O, Sr­(NO_3_)_2_, or Cr­(NO_3_)_3_·9H_2_O was dissolved directly in an aqueous solution with Mn­(NO_3_)_2_·4H_2_O and Ni­(NO_3_)_2_·6H_2_O at stoichiometric ratios, and the concentration of all dopants in the resulting LNMO cathode materials was fixed at 0.1 per formula unit.

For studies on the effect of doping level, the same procedure applied to the dopant screening study was used to prepare the Ca-doped LiNi_0.5_Mn_1.5–x_Ca_*x*_O_4_ (x = 0.01, 0.03, 0.05, 0.07, and 0.1) cathode materials, except various amounts of Ca­(NO_3_)_2_·4H_2_O added to the metal solution to achieve Ca concentrations of 0.01, 0.03, 0.05, 0.07, and 0.1 per formula unit in LNMO.

### 2.3 Material Characterizations

#### 2.3.1 Crystallinity

The crystal structure of the LNMO was characterized by using an X-ray diffractometer (XRD, Rigaku SmartLab SE) with Cu Kα radiation (λ = 1.5406 Å).

#### 2.3.2 Morphology and Microstructure

The morphological information on the obtained materials was collected using a scanning electron microscope (SEM, JEOL IT200, accelerating voltage range 1–30 kV). High-resolution transmission electron microscopy (HRTEM) imaging and selected area electron diffraction (SAED) of the LNMO particles were performed using a Thermo Fisher Talos F200X G2 S/TEM instrument.

#### 2.3.3 Composition

The compositions of the undoped and doped materials were analyzed by inductively coupled plasma optical emission spectroscopy (ICP-OES; Agilent 5110). X-ray photoelectron spectroscopy (XPS, Thermo Scientific ESCALAB 250Xi) with Al Kα radiation was also performed to analyze the surface chemical states and elemental composition of the samples. To determine the Ca distribution within the particles, an electron microscope (SEM) equipped with energy-dispersive X-ray spectroscopy (EDS) and electron probe microanalysis (EPMA) was used to examine the LNMO particles with cross-sections exposed. For cross-sectional analysis, the LNMO powders were embedded in epoxy resin and kept at room temperature overnight until they were fully cured. The cured samples were sliced, ground, and polished to expose smooth cross-sections of the particles.

### 2.4 Electrode Fabrication and Cell Assembly

#### 2.4.1 Slurry Preparation

Typically, the active material of LNMO powders and the conductive additive of C45 carbon black powders were first mixed and then dispersed in a binder solution with 8 wt % PVDF dissolved in NMP. Additional NMP was added to adjust the viscosity, and the total solid content of the slurry was approximately 40 wt %. The slurry was uniformly cast onto aluminum foil using a doctor blade and initially dried at 40 °C for 4 h to allow gradual evaporation of NMP. The electrodes were then transferred to a vacuum oven and further dried at 120 °C overnight to ensure complete removal of residual NMP. All dried electrodes had the same composition: 80 wt % active material, 10 wt % conductive additive, and 10 wt % binder.

#### 2.4.2 Cell Assembly

The dried electrodes were punched into 9/16 in. disks and assembled in CR2032-type coin cells with lithium metal foil as counter electrodes. The solution composed of 1.2 M LiPF_6_ in ethylene carbonate (EC)/ethyl methyl carbonate (EMC) (3/7 by weight) was used as the electrolyte. All cell assemblies were conducted inside an Ar-filled glovebox.

### 2.5 Electrochemical Evaluations

Galvanostatic charge/discharge cycling was performed in the voltage range of 3.5–4.95 V vs Li/Li^+^ on a Neware BTS-4000 battery testing system at room temperature. All cells underwent three initial formation cycles at 0.1 C, assuming the specific capacity at 1 C was 147 mAh/g, to stabilize the electrode/electrolyte interface. After the formation test, the rate capability test was conducted with a constant current density of 0.2 C during the charge process, and the current density varied from 0.2 C to 0.5 C, 1 C, 2 C, 5 C, and 10 C during the discharge process. By the end of the rate test, a recovery test at 0.2 C was applied. At each rate, the cells underwent three cycles. After the formation test, long-term cycling was conducted with a constant current rate of 0.5 C applied to both the charge and discharge processes.

Electrochemical impedance spectroscopy (EIS) was performed on a Gamry Interface 1010E electrochemical workstation with an excitation amplitude of 5 mV over a frequency range of 10^6^ to 5 × 10^–2^ Hz. Before impedance measurements, all cells underwent three formation cycle tests and were charged to 50% capacity. Cyclic voltammetry (CV) measurements were conducted in the potential range of 3.5–5.0 V vs Li/Li^+^ at a scan rate of 0.1 mV/s using a Biologic VSP multichannel potentiostat.

GITT was performed to evaluate the state-of-charge (SOC)-dependent Li^+^ transport kinetics in LNMO half-cells. Small galvanostatic current pulses (0.1 C) followed by open-circuit relaxation were applied over 3.5–4.95 V vs Li/Li^+^, and $D_{Li^{+}}$ was calculated using the standard Weppner–Huggins approach. Because GITT-derived $D_{Li^{+}}$ is sensitive to the assumed diffusion length in composite electrodes, identical analysis assumptions were used for all samples to enable reliable relative comparison between Ca-doped and undoped LNMO. SOC was defined as SOC = 100% at the fully charged (delithiated) state and SOC = 0% at the end of the discharge cutoff.

## 3 Results and Discussion

### 3.1 Dopant Screening

Three elements, Ca, Sr, and Cr, were preliminarily investigated as dopants in LNMO cathodes to verify the hypothesis that dopants with strong metal–oxygen affinity can enhance lattice oxygen stability, suppress oxygen loss during cycling, and identify the optimal dopant with a large ion size. Figure S1 demonstrates the cycling performance of LNMO cathode materials doped with different elements. Regardless of the dopants, all doped LNMO samples exhibited capacity retentions higher than, or at least comparable to, that of the baseline LNMO cathode without dopants (Figure S1b). Among all four samples, Ca-doped LNMO exhibited the highest capacity retention (∼94%) after 500 cycles, and the initial discharge capacity (∼124 mAh/g) was almost identical to that of the dopant-free baseline (Figure S1a). In contrast, Sr- and Cr-doped LNMO cathodes deliver initial capacities of ∼111 and ∼119 mAh/g, respectively, which are lower than that of the baseline. However, Sr-doped LNMO demonstrated improved capacity retention (∼87%) after 500 cycles, higher than those of Cr-doped and dopant-free baseline LNMO cathodes (∼84% capacity retained after 500 cycles). The electrochemical performance of doped LNMO cathodes might be associated with the ionic radii, charge, and strength of M–O bonds, which are summarized in Table S1. Among all dopants, Ca^2+^ exhibits a moderate ionic radius (1.00 Å, coordination number (CN) = 6) and a relatively strong Ca–O bond (∼464 kJ/mol) and moderate ionic potential (z/r = 2). This provides a favorable balance between lattice stabilization and minimal lattice strain caused by structural distortion in the presence of large doping ions. In contrast, the significantly large ionic radius (1.18 Å, CN = 6) and lower ionic potential (z/r ≈ 1.70) of Sr^2+^ may cause lattice distortion or even destroy the oxygen framework in the crystal structure, leading to poor cycling performance of doped LNMOs. In addition, electron transport usually occurs through the M–O–M pathways formed by the overlapped transition metal 3d and oxygen 2p orbitals. The substitution of Ni/Mn by too large Sr atoms might also disrupt this pathway, reduce effective electron hopping and overall conductivity, and thus lower the capacity of the Sr-doped LNMO cathode.^19^ Although Cr has the smallest ionic radius among these three dopants, its trivalent nature is often accompanied by the formation of oxygen vacancies when it replaces Ni/Mn in cathode materials.^20,21^ Each oxygen vacancy can reduce the neighboring Mn^4+^ to Mn^3+^, thereby increasing the Mn^3+^ content in the spinel structure.^2,22,23^ The high Mn^3+^ content will exacerbate the Jahn–Teller distortion in the LNMO crystal and, therefore, lower the cycling stability of cells. In light of its excellent electrochemical performance, Ca was, therefore, selected as the dopant for the LNMO cathode in the following investigations on the effect of doping level.

### 3.2 Effect of Ca Doping on the Structural Properties and Electrochemical Performance of LNMO

The incorporation of Ca into the crystal structure of doped LNMO can be confirmed from ICP-OES data (Table S2). The ICP data demonstrate a relatively accurate elemental ratio for each component in the absence of Ca in the undoped LNMO sample. The Ca element can be found in the Ca 0.05 sample, and the presence of Ca merely lowers the molar ratio of Mn. This implies that the majority of Ca primarily substitutes partial Mn, rather than Ni, in the doped LNMO sample. The incorporation of the Ca dopant into the LNMO particles can be validated by the analysis of EDS elemental mapping and line profiles collected from the Ca 0.05 LNMO sample. Figure 1 reveals a uniform distribution of Ni, Mn, O, and Ca across the whole cross-section of an individual Ca 0.05 LNMO particle. EDS (Figure 1d) and EPMA (Figure S2) line scans on the cross-sectional area of LNMO further confirm that Ca is evenly distributed throughout the particle, and no aggregation of Ca is observed. The incorporation of Ca will result in a Mn^3+^ content change in the LNMO cathode, which can be corroborated by XPS analysis of the Mn 2p spectra (Figure 2). In the Ca 0.05 sample, the Mn 2p_3/2_ spectrum exhibits a diminished Mn^3+^ component at ∼641.8 eV and a stronger Mn^4+^ peak at ∼642.9 eV compared to undoped LNMO, leading to a higher Mn^4+^/Mn^3+^ ratio. A similar trend is observed for the 2p_1/2_ doublet, confirming that Ca doping increases the average Mn oxidation state and suppresses the Mn^3+^ content.

**Figure 1:**
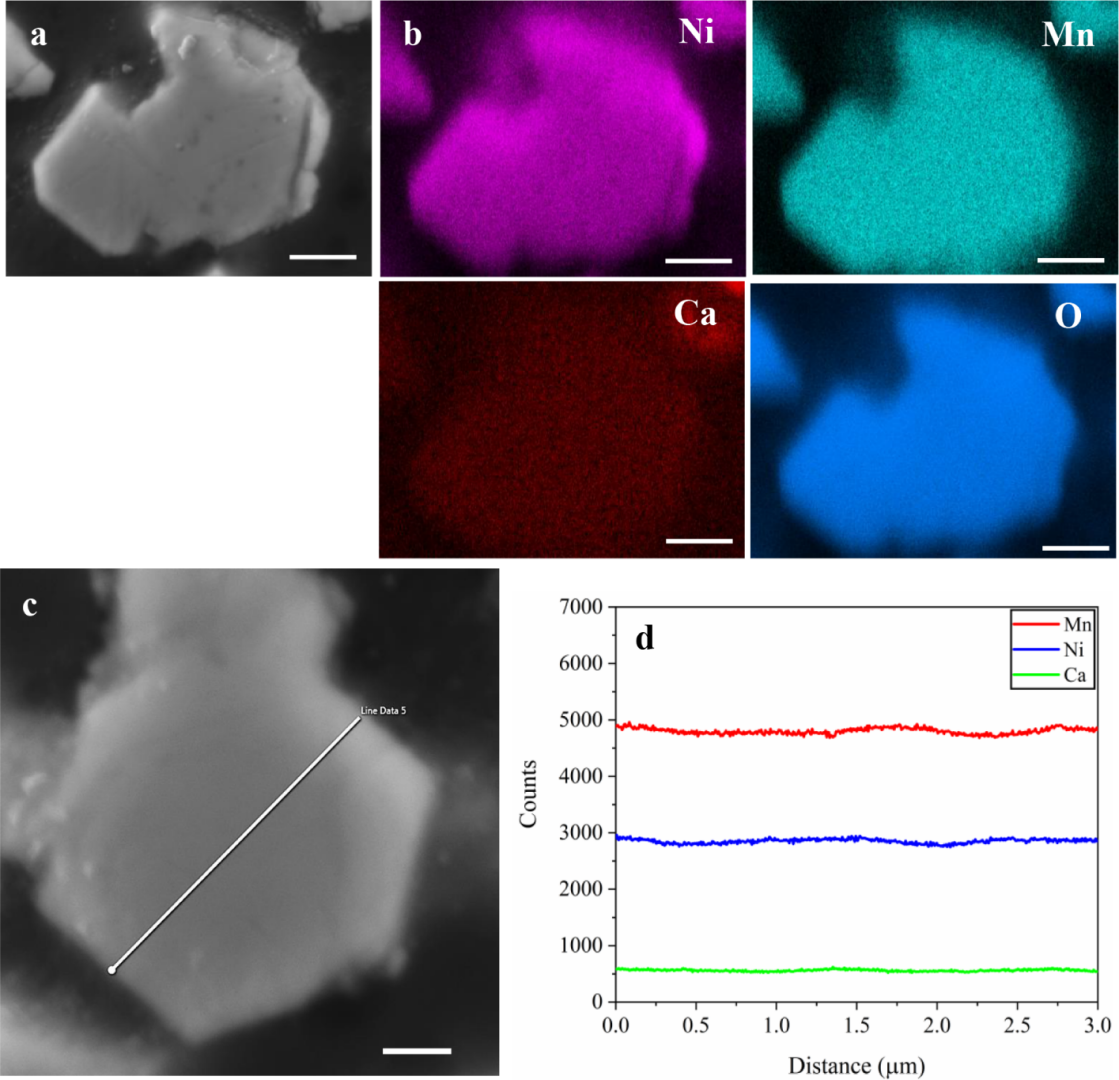
(a, c) Cross-section SEM images. (b) EDS elemental mapping. (d) EDS line profiles for Ni, Mn, and Ca along the marked line in the SEM image of the Ca 0.05-doped LNMO particle. The scale bars represent 1 μm.

**Figure 2:**
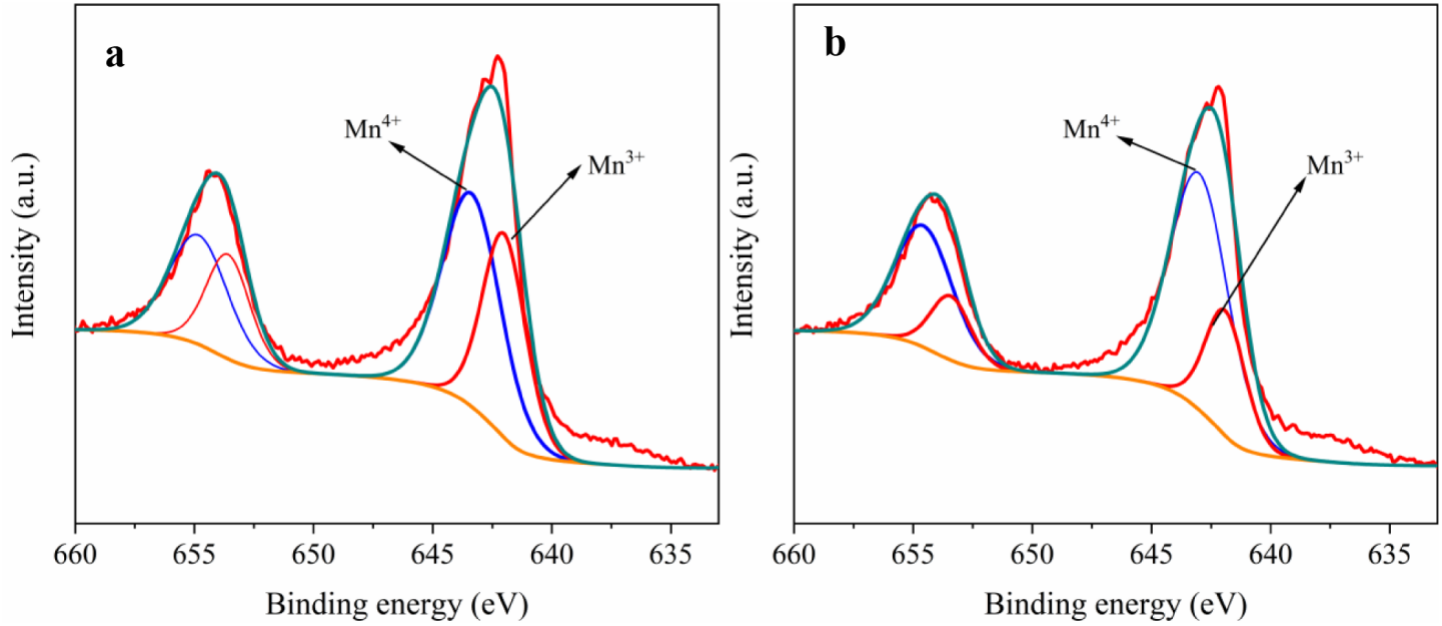
XPS spectra of (a) undoped and (b) Ca 0.05 LNMO samples.

The introduction of Ca can effectively modify the structure of the LNMO crystals. Figure 3 shows the XRD patterns of undoped and Ca-doped LNMO samples at various doping levels. The diffraction peaks observed from all samples can be identified as the characteristic reflections of the spinel LNMO phase with the *Fd*3̅*m* space group, and no peaks corresponding to impurities or other crystal phases are observable. This indicates that Ca doping does not greatly modify the primary structure of the LNMO cathode material, even for the Ca 0.1 sample, with the highest doping level in this study. However, the (400) peak shifts to lower angles with increasing doping levels (Figure 3b). The peak shift implies the incorporation of Ca, which has an ionic radius of 1.00 Å larger than those of Ni^2+^ (0.69 Å) and Mn^4+^ (0.53 Å), into the crystal lattice, leading to an increase in the lattice parameter.^24^ Consequently, the unit cell volumes slightly increase with the doping level. The lattice parameters calculated by Rietveld refinement are summarized in Table S3, and the corresponding refinement profiles are displayed in Figure S3. With the Ca doping level enhanced from 0 to 0.1, the lattice parameter of the LNMO sample increases almost linearly from 8.168 Å (undoped) to 8.194 Å (Figure 3d), which perfectly obeys Vegard’s law stating that the lattice constant of an alloy or solid solution varies linearly with its composition.^1,24^ This implies that Ca ions are successfully incorporated into the LNMO lattice rather than forming secondary phases. More information about the effect of the Ca doping level on the LNMO crystal structure can be revealed through the analysis of the relative peak intensity ratios, which are summarized in Table S4. The relative intensity ratio of I(400)/I(111) peaks decreases with the increase in the Ca doping level, reaches a minimum value at 0.05, and increases with further increases in the doping level. It should be noted that for powder XRD, relative peak intensities can be influenced not only by preferred orientation but also by changes in structure factors resulting from Ni/Mn cation ordering. Therefore, the I(400)/I(111) ratio here is a qualitative indicator rather than standalone proof of facet dominance. Notably, the Ca 0.05 sample that exhibits the minimum I(400)/I(111) ratio also shows the most uniform sharp-edged octahedral morphology in SEM (Figure 4d), which is consistent with LNMO particles with preferential growth of {111} planes.^25^ At higher Ca content (x = 0.1), the I(400)/I(111) ratio increases again, consistent with the increased ratio of {100} facets. Since the {100} surface planes, represented by the (400) reflection, are thermodynamically less stable and associated with higher surface energy than {111} planes, a reduced I(400)/I(111) ratio suggests preferential growth of the low-energy {111} surface planes in the Ca 0.05 sample.^26–29^ In addition, the I(111)/I(311) ratio increases from 1.94 for undoped LNMO sample gradually to 3.14 for Ca 0.1 sample, suggesting that elevating the doping level can effectively improve the cation ordering (less cation mixing) in Ca-doped LNMO cathodes.^23,26,30^ The I(311)/I(400) ratios of all samples are in the range of 0.96–1.10, implying the structural stability of LNMO cathodes prepared via the coprecipitation route in this work.

**Figure 3:**
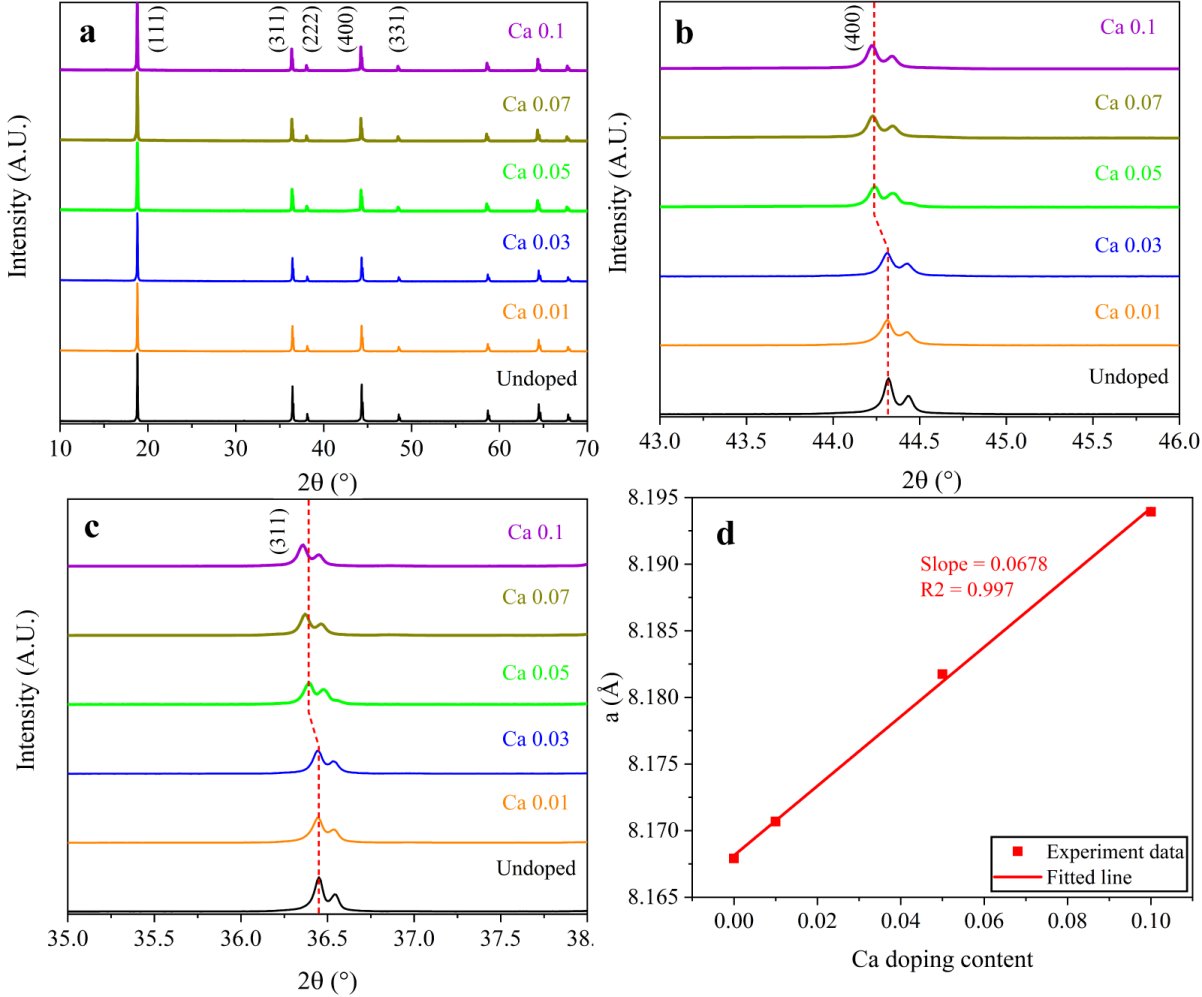
(a) Full-range X-ray diffraction (XRD) patterns of undoped and Ca-doped LNMO samples. (b) Enlarged view of the 2θ range 43°–46°. (c) Enlarged view of the 2θ range 35°–38°. (d) Refined lattice parameter a as a function of Ca doping content in LNMO cathode materials.

**Figure 4:**
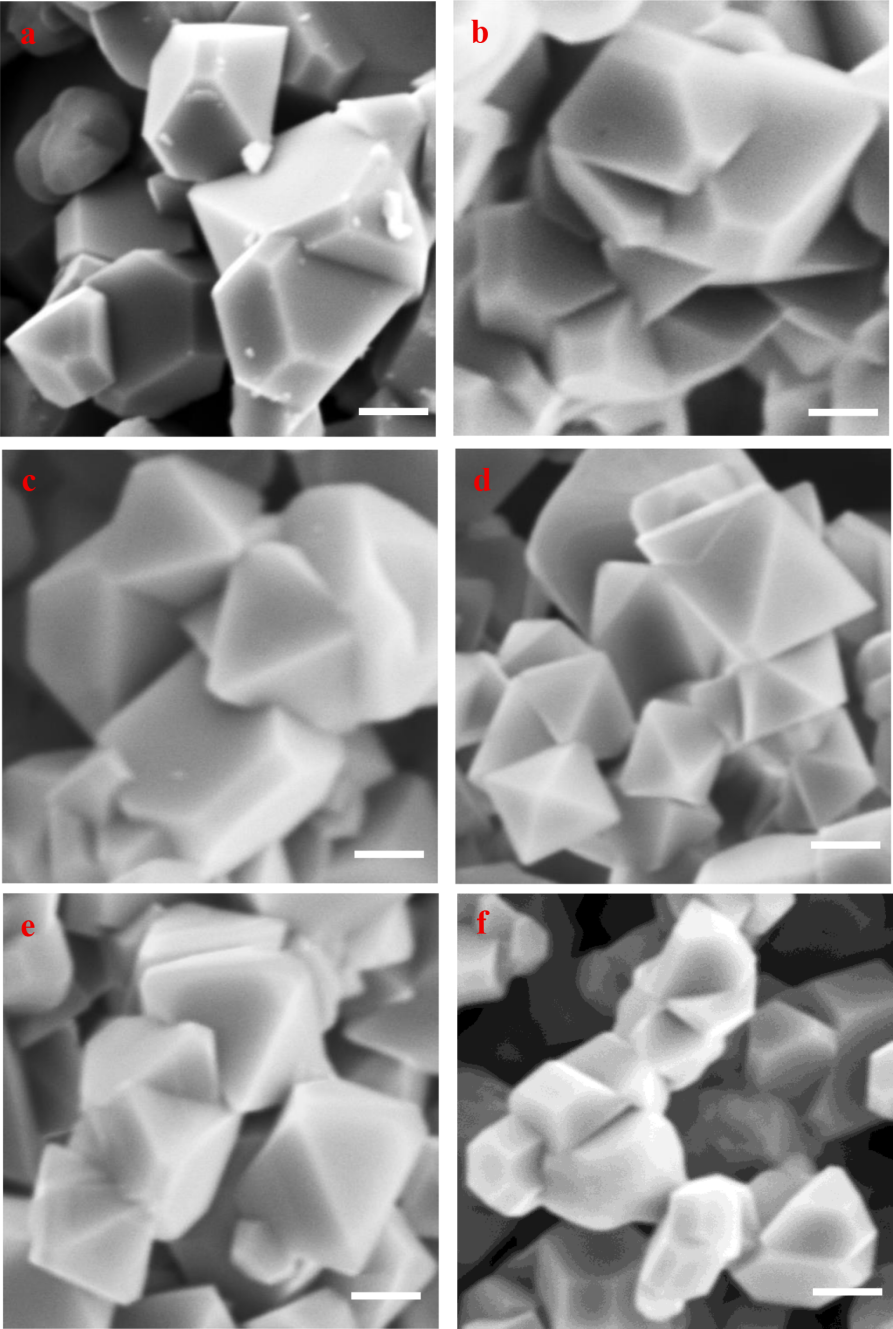
SEM images of (a) undoped, (b) Ca 0.01, (c) Ca 0.03, (d) Ca 0.05, (e) Ca 0.07, and (f) Ca 0.1 LNMO cathodes. All scale bars are 1 μm.

The effect of Ca doping on the morphology of the LNMO cathode crystal can be found from SEM observations. Figure 4a shows that, in the absence of a Ca dopant, the undoped LNMO sample is composed of numerous particles mainly exhibiting a truncated octahedral morphology, and a few spherical particles are also observable. This implies the simultaneous growth of both {100} and {111} surface facets in the dopant-free LNMO crystal. In addition, the particle size of the baseline LNMO sample is distributed within a relatively wide range of 1–4 μm. The introduction of a Ca dopant can modify not only the particle morphology but also the size distribution. At the lowest doping level, the Ca 0.01 sample particles still show a truncated octahedral shape but with less {100} surface facet exposure, compared to those in the undoped LNMO particles (Figure 4b). With an increase in the Ca doping level, fewer {100} planes are exposed (Figure 4c) and the Ca 0.05-doped LNMO exhibits a well-defined octahedral morphology with sharper edges (Figure 4d), suggesting a preferential growth of the more stable {111} surface facets. In addition, the Ca 0.05 sample is composed of relatively uniform particles with sizes distributed in a narrow range of 1–2 μm. Further increasing the Ca doping level (up to Ca 0.1) results in the reoccurrence of {100} planes, with more LNMO particles exhibiting a truncated octahedral shape (Figure 4e and f). The SEM observations of the morphological changes are consistent with the XRD results which show that the Ca 0.05 sample demonstrates minimal {100} surface facet exposure, while doping levels above or below 0.05 mol Ca lead to more {100} exposure in the LNMO crystals (Table S4). It is well recognized that {111} surface facets have the lowest surface energy due to their densely packed atomic arrangement and thus the highest surface stability compared to other planes.^25,26^ Thus, it is rational to expect excellent electrochemical performance from the Ca 0.05 cathode.

The effect of Ca doping on the microstructure of the LNMO crystal can be observed from the TEM observations (Figure 5). From the HRTEM image, continuous lattice fringes were observed in an undoped LNMO particle (Figure 5a), indicative of individual and single-crystalline particles. The distance between adjacent lattices was measured to be about 2.579 Å, which corresponds to the {310} planes of the cubic structure (*Fd*3̅*m*). The electron diffraction (ED) of the undoped LNMO sample clearly demonstrates discrete and patterned spots (inset, Figure 5a), indicating the excellent integrity of the single crystals. All diffraction spots can be indexed by assuming cubic systems with *Fd*3̅*m* symmetry. The HRTEM observation on Ca 0.05 LNMO (Figure 5b) revealed well-defined lattice fringes that could be assigned to the {111} planes of the spinel lattice with an interplanar spacing of ∼4.723 Å. The particle exhibited a coherent single-crystal domain in the fringe pattern, indicating preserved crystallinity after Ca doping. The associated ED pattern (inset, Figure 5b) showed discrete, evenly distributed spots that match the cubic *Fd*3̅*m* symmetry. This confirms that at the 0.05 doping level, the bulk structure remains fully disordered, in line with the XRD results showing no evidence of cation ordering (Figure 3). Meanwhile, Ca 0.1 LNMO with the highest doping level here exhibits a different structural feature. The lattice fringes are continuous and correspond to the {111} planes (Figure 5c), confirming that particles maintained a single-crystalline nature similar to that of undoped and Ca 0.05 LNMO samples. However, the ED pattern differs from those of baseline and Ca 0.05 LNMO samples by displaying additional faint reflections in addition to the cubic spots (inset, Figure 5c). These weak superlattice spots indicate the emergence of partial Ni/Mn ordering at elevated Ca content.^30^ Such ordering is consistent with the rise in the I(111)/I(311) intensity ratio observed in XRD, reflecting the structural transition from a fully disordered to a partially ordered spinel state. The occurrence of ordering at this higher Ca doping level (Ca 0.1 LNMO) introduces lattice strain, which has been correlated with reduced transport kinetics despite preserved crystallinity.^11^ Furthermore, TEM-EDS elemental mapping confirms that Ni, Mn, O, and Ca are homogeneously distributed across the particle, with no evidence of Ca-rich domains at the particle surfaces (Figure S4). Based on the observations from XRD and electron microscopies, it could be concluded that the Ca element was successfully integrated into the LNMO crystal structure through the doping method and uniformly distributed across an individual single-crystal particle.

**Figure 5:**
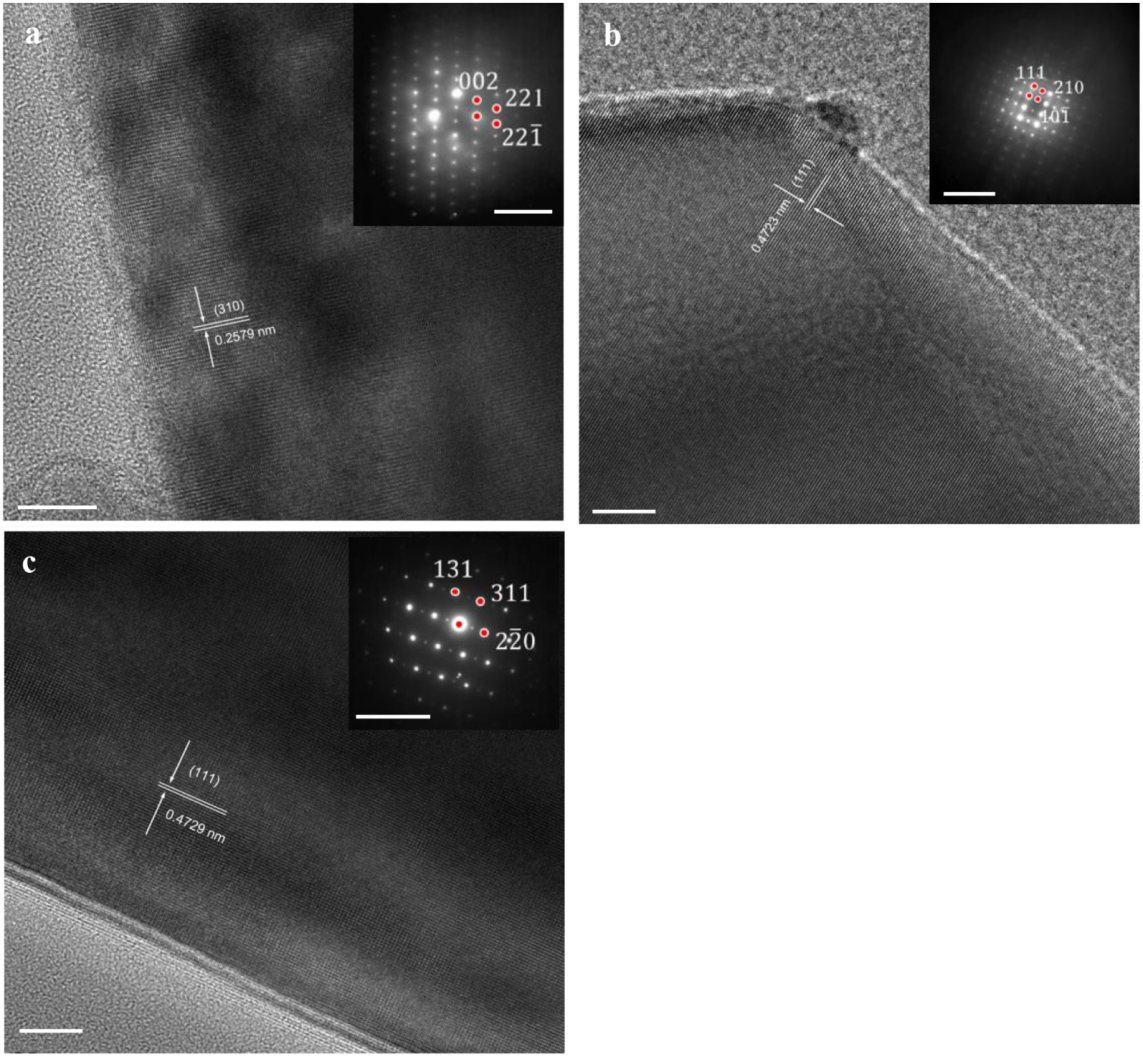
HRTEM images of (a) undoped, (b) Ca 0.05, and (c) Ca 0.1 LNMO cathodes. Insets show electron diffraction. Scale bars in HRTEM represent 10 nm, and 10 nm^–1^ for insets.

It is well recognized that electrochemical performance strongly depends on the microstructure and surface properties of LNMO particles. From XRD and SEM, the Ca 0.05 cathode exhibits the minimum I(400)/I(111) together with a well-defined octahedral morphology, indicating suppression of high-energy {100} facets and preferential exposure of densely packed {111} facets. The preferential growth of surface {111} facets reduces parasitic surface side reactions, thereby lowering polarization during cycling.^26^ XRD and TEM-SAED results further confirm that both undoped and Ca 0.05 LNMO cathodes maintain a disordered spinel framework without superlattice reflections, while the Ca 0.1 LNMO cathode shows proof of partial ordering. It has been widely reported that disordered LNMO exhibits superior electrochemical properties compared with the ordered phase, as the solid solution lithiation pathway reduces lattice strain and voltage hysteresis, whereas ordered LNMO tends to undergo two-phase reactions that generate mechanical stress and hinder Li^+^ transport.^25,30^ The disordered structure at Ca 0.05, combined with a moderate Mn^3+^ content that mitigates Jahn–Teller distortion, promotes a stable bulk structure during cycling. It is, therefore, expected that the Ca 0.05 cathode would exhibit superior electrochemical performance due to the synergistic effects of enriched {111} facet exposure, preserved cation disorder, and balanced Mn^3+^ concentration, which together suppress interfacial side reactions, enhance Li^+^ transport, and sustain long-term structural integrity. Similar reports have shown that a moderate concentration of Mn^3+^ in LNMO is beneficial for cycling stability.^25,30^

The advantages of the Ca dopant can be validated by the excellent electrochemical performance of LNMO cathodes. Figure 6a shows that, regardless of the doping level, all LNMO cathodes exhibit three distinct plateaus, a lower voltage plateau at ∼4.0 V, associated with the Mn^3+^/Mn^4+^ redox reaction, and two high voltage plateaus in the 4.6 V–4.8 V region, corresponding to Ni^2+^/Ni^3+^ and Ni^3+^/Ni^4+^ redox reactions, during the charge/discharge process. The Ca dopant has little effect on the high voltage plateaus but notably affects the low voltage plateau. The relative capacity contribution from the 3.8 to 4.2 V plateau can be used to quantify the Mn^3+^ content in the spinel lattice, and the corresponding capacity contribution for each sample is summarized in Table S5. Among all samples, the undoped LNMO cathode demonstrates the longest plateau at the low voltage of ∼4.0 V, and it becomes shorter as the Ca doping level increases. The shorter plateau at the low voltage indicates an increase in the average Mn oxidation state and mitigation of the Jahn–Teller effect upon Ca doping.^30^ In addition, the shorter low voltage plateau implies less electrochemically active Mn^3+^ involved in the redox reaction in the presence of electrochemically inactive Ca atoms. This can be confirmed by the effect of the Ca dopant on the specific capacity delivered by LNMO cathodes. In the absence of the Ca dopant, the undoped LNMO can deliver a specific capacity of ∼131 mAh/g. As the Ca doping level increases, the discharge capacity gradually decreases to ∼130, 129, 126, 124, and 121 mAh/g for Ca 0.01, Ca 0.03, Ca 0.05, Ca 0.07, and Ca 0.1 samples, respectively (Table S5). The progressive suppression of the 3.8–4.2 V plateau with Ca is consistent with a higher average Mn valence (less Mn^3+^) observed from XPS analysis (Figure 2) and reduced surface side reactions associated with oxygen vacancies. This combined effect arises through the substitution of transition metal cations with Ca^2+^ in the LNMO lattice, which promotes charge compensation by converting Mn^3+^ to Mn^4+^. Meanwhile, the strong Ca–O bonds suppress oxygen-vacancy formation and enhance the structural stability of the spinel framework during cycling.

**Figure 6:**
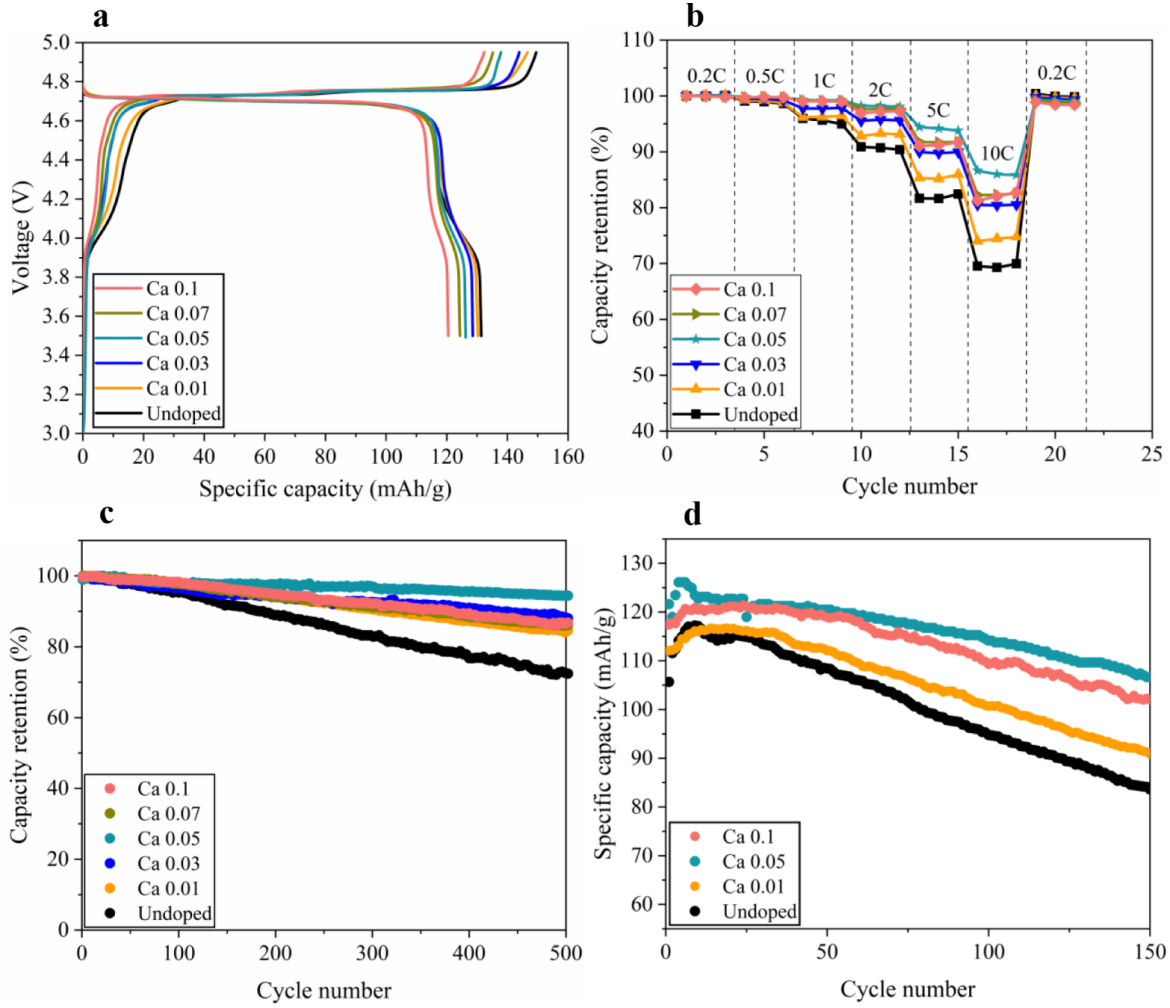
Electrochemical performance of Ca-doped LNMO cathodes: (a) voltage profiles during the third formation cycle. (b) Rate capability at 25 °C. Specific capacity is normalized to the first cycle. (c) Cycling performance at 25 °C. (d) Cycling performance of undoped and Ca 0.05 LNMO at 55 °C.

The Ca doping can effectively improve the rate capability of the LNMO cathodes. Figure 6b and Figure S5 show that regardless of the doping level, the specific capacity delivered by all LNMO cathodes decreases when the current density increases. At every current density, Ca-doped LNMO cathodes exhibit superior capacity retention over the undoped samples, and among all doped samples, the Ca 0.05 cathode sample shows the highest rate capability. Under the low current densities of ≤2 C, the Ca 0.05 cathode has a capacity retention of >99%, which decreases gradually to ∼98% at 5C and finally reaches ∼90% at 10 C. Lowering the Ca doping level greatly decreases the rate capability of LNMO cathodes. The undoped LNMO cathode retains a capacity retention of ∼95% at 1 C, which decreases to ∼90% at 2 C, ∼82% at 5 C, and ∼70% at 10 C. Enhancing the Ca doping level slightly decreases the rate capability of LNMO cathodes. Under the low current densities of ≤2C, the Ca 0.1 cathode also exhibits >99% capacity retention, and it decreases slightly to ∼93% at 5C and ∼82% at 10 C. In addition, the undoped LNMO cathode delivers the highest specific capacity of ∼132 mAh/g at 0.2 C. The specific capacity delivered by Ca-doped LNMO samples at the low current density of 0.2 C decreases when the Ca doping level increases. The specific capacity decreases to ∼130 mAh/g for the Ca 0.03 sample, 128 mAh/g for the Ca 0.07 sample, and eventually 123 mAh/g for the Ca 0.1 sample. At high current densities of ≥2C, however, the Ca 0.05 delivers the highest capacities of ∼128, ∼121, and ∼111 mAh/g at 2 C, 5 C, and 10 C, respectively. Meanwhile, the undoped LNMO shows the lowest specific capacities when the current densities are above 2 C.

To further elucidate the origin of the improved high-rate behavior, representative galvanostatic voltage profiles at selected C-rates are compared in Figure S6. Compared to the undoped LNMO, Ca 0.05 exhibits reduced voltage polarization at all rates, and the discharge plateau remains well defined even at a high rate of 10 C. The reduced polarization indicates lower kinetic overpotential during high-rate operation, consistent with the higher discharge capacities of Ca 0.05 at ≥2C (Figure 6b and Figure S5). Similar relationships between reduced voltage polarization and enhanced rate capability have been reported for doped LNMO systems, where improved reaction kinetics and suppressed impedance growth help preserve accessible capacity at high current densities.^31^

The superior rate capability might be associated with the increased ionic diffusion in the Ca-doped cathodes. Figure 7a shows almost overlapping voltage profiles for undoped and Ca 0.05 at the low current density of 0.2 C. When the current density was enhanced to 2 C, the undoped LNMO cathode demonstrated higher polarization than that of the Ca 0.05 cathode, and this difference became more prominent at 10 C. The reduced cell polarization might be associated with the lower resistances of Ca-doped LNMO cathodes. This can be corroborated by the analysis of electrochemical impedance spectroscopy (EIS) data collected on cells with Ca-doped LNMO electrodes (Figure 7b). All LNMO samples exhibit two compressed semicircles followed by a straight line in the low-frequency region. The high-frequency semicircle is associated with the resistance of the cathode–electrolyte interphase (R_CEI_), while the medium-frequency semicircle corresponds to the charge transfer resistance (R_CT_). The inclined tail is characteristic of Warburg impedance arising from lithium ion diffusion. Ca 0.05 exhibits the lowest combined semicircles among all samples, consistent with its reduced polarization and superior rate performance. The improved interfacial kinetics of Ca 0.05 can be attributed to its smaller particle size, which shortens Li^+^ diffusion paths. In addition, the Ca substitution enhances the structural integrity of the spinel structure by forming stronger Ca–O bonds, which are more robust than Mn–O or Ni–O bonds.^32^ In contrast, undoped LNMO exhibits the largest semicircles among all samples, in agreement with its low rate capability. Excessive Ca doping (Ca 0.1) also increases the interfacial resistance compared to Ca 0.05, suggesting that excessive Ca doping may disrupt Li^+^ transport pathways and undermine Li diffusion. To further support these findings, the lithium diffusion coefficients $\left({D_{\mathrm{Li}}}^{+}\right)$ (Figure 7c) were calculated from the Warburg region of EIS spectra according to the method reported previously.^33^ Ca 0.05 exhibits the highest ${D_{\mathrm{Li}}}^{+}$ of 1.134 × 10^–12^ cm^2^ s^–1^, significantly higher than that of the undoped LNMO (2.273 × 10^–13^ cm^2^ s^–1^). In contrast, further increasing the Ca content from 0.05 to 0.1 leads to a reduction in ${D_{\mathrm{Li}}}^{+}$ . These calculation results correlate well with the rate capability results and Nyquist plots. Overall, the EIS results support the conclusion that moderate Ca doping (Ca 0.05) can enhance interfacial kinetics, lower electrode polarization, and improve the rate capability of LNMO.

**Figure 7:**
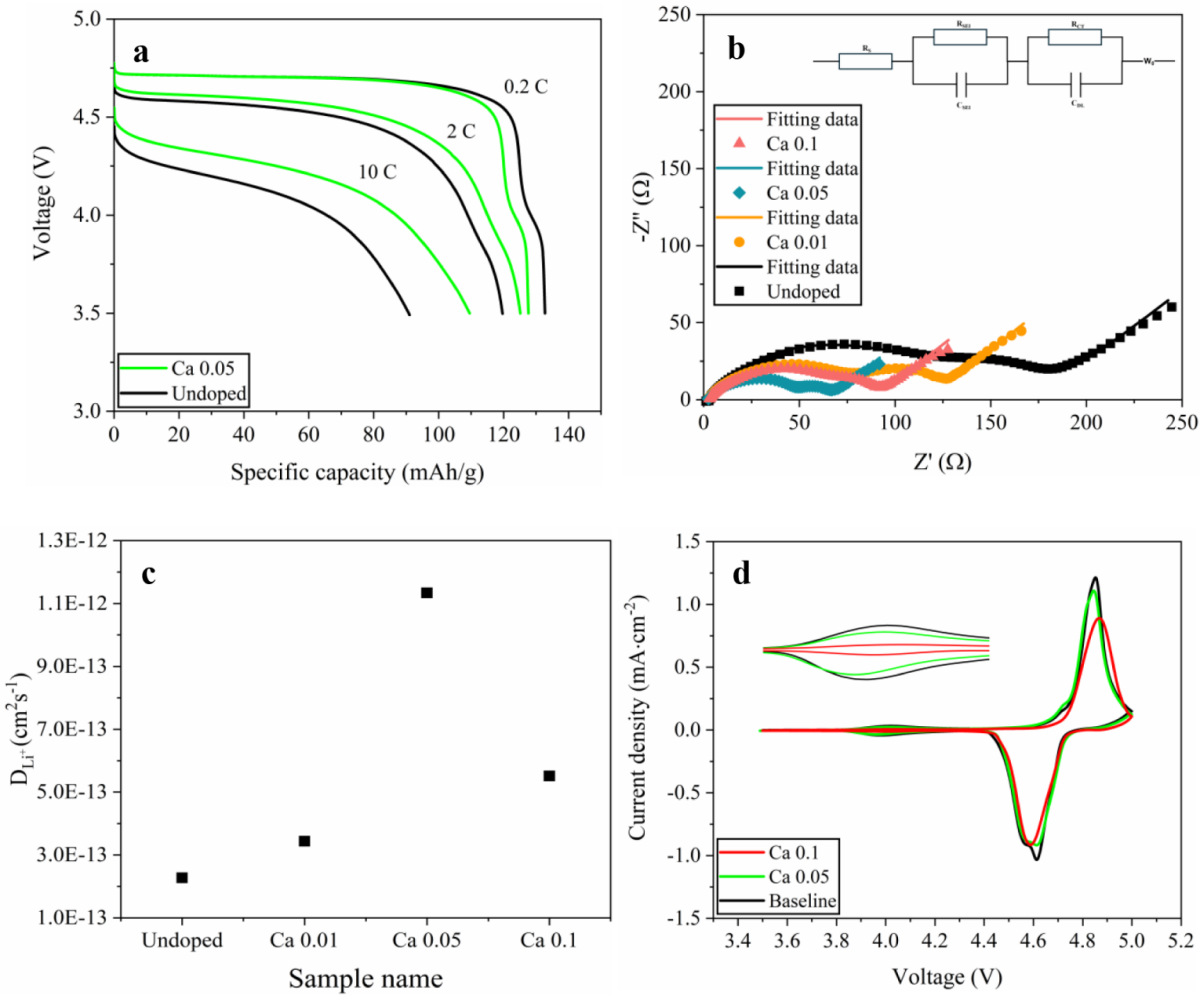
(a) Discharge voltage profiles of undoped and Ca 0.05 LNMO at different rates. (b) Representative EIS Nyquist plots of Ca-doped LNMO electrodes with fitted curves. Inset shows the equivalent circuit for fitting. (c) Li^+^ diffusion coefficients $\left(D_{Li^{+}}\right)$ calculated from the Warburg region of the EIS spectra. (d) CV curves of undoped and Ca-doped LNMO collected at a scan rate of 0.2 mV/s. Inset highlights the 3.8–4.2 V region associated with the Mn^3+^/Mn^4+^ redox couple.

GITT was further employed to investigate bulk Li^+^ transport as a function of SOC (Figure S7). The obtained $D_{Li^{+}}$ values fall within the typical range reported for LNMO (≈10^–12^–10^–8^ cm^2^ s^–1^). $D_{Li^{+}}$ exhibits significant SOC dependence, with reduced values observed in the mid-SOC region and higher diffusion coefficients at low and high SOC. Such nonmonotonic behavior is widely reported for LNMO and is commonly associated with plateau or two-phase regions near the Ni^2+^/Ni^4+^ redox potential, where the small voltage change with respect to lithium content reduces the apparent chemical diffusion coefficient derived from GITT.^34^ Under identical analysis assumptions, Ca 0.05 exhibits higher $D_{Li^{+}}$ than undoped LNMO over most SOC, consistent with its improved Li^+^ transport. Notably, the absolute $D_{Li^{+}}$ values derived from GITT differ from those obtained by EIS. This discrepancy is expected because GITT yields a chemical diffusion coefficient based on transient polarization and quasi-equilibrium potential shifts under finite perturbation, whereas EIS probes small signal diffusion within a porous electrode framework.^35,36^ Differences in effective diffusion length, active surface area, and semi-infinite diffusion assumptions can therefore lead to order-of-magnitude variation between the two techniques.

Redox behavior and polarization of the electrodes can provide further insight into the reaction kinetics and reversibility of LNMO. Cyclic voltammetry (CV) was carried out on Ca-doped and undoped LNMO (Figure 7d). All electrodes exhibit the characteristic high-voltage redox couples of LNMO: two anodic peaks in the ∼4.7–4.8 V region assigned to the Ni^2+^/Ni^3+^ and Ni^3+^/Ni^4+^ transitions, respectively, and a weak anodic peak at ∼4.0 V corresponding to the Mn^3+^/Mn^4+^ transformation. Ca 0.05 exhibits a smaller anodic–cathodic separation for the Ni redox couples than that of the undoped LNMO, indicating lower polarization after Ca doping. The ∼4.0 V Mn^3+^ anodic peak also becomes weaker as the Ca content increases, consistent with the reduced Mn^3+^ content as the Ca content increases based on formation analysis (inset in Figure 7d and Table S5). By comparison, undoped LNMO displays a larger anodic–cathodic separation than Ca 0.05, indicating a larger polarization. Meanwhile, the two distinct redox peaks corresponding to the Ni^2+^/Ni^3+^ and Ni^3+^/Ni^4+^ couples can be observed in both Ca 0.05 and undoped LNMO, whereas these peaks merge into a single broadened peak in Ca 0.1 LNMO. The increased hysteresis and merged Ni redox peaks are consistent with previous reports that more ordered LNMO exhibits larger polarization, less distinct Ni^2+^/Ni^3+^ and Ni^3+^/Ni^4+^ redox processes, and inferior kinetic reversibility compared with disordered LNMO.^23,25^ These results align well with the TEM-SAED observations, where Ca 0.1 shows superlattice reflections characteristic of ordering, while undoped and Ca 0.05 remain disordered with no superlattice (Figure 5).

The Ca doping level also plays a significant role in determining the cyclability of LNMO cathodes. Figure 6c shows that the initial capacities of all LNMO cathodes are in the range of 120–130 mAh/g, and the undoped LNMO exhibits the fastest capacity decay, retaining only ∼96 mAh/g (∼73% of the initial capacity) after 500 cycles at room temperature. In contrast, all Ca-doped LNMO cathodes demonstrate significantly improved cyclability with >85% of the initial capacity retained after 500 cycles. Especially, the Ca 0.05 cathode delivers the highest cycling retention, maintaining ∼121 mAh/g (∼94% of the initial capacity) after 500 cycles. To study the stability of LNMO cathodes at high temperatures, we also conducted cycling tests on undoped LNMO and Ca 0.05 cathodes at 55 °C. Figure 6d shows that the specific capacity delivered by undoped LNMO drops dramatically from ∼118 mAh/g to ∼83 mAh/g (∼70% capacity retention) within 150 cycles. In contrast, all Ca-doped samples demonstrate improved stability at an elevated temperature. Among them, the Ca 0.05 LNMO delivers the highest initial capacity (∼125 mAh/g) and maintains ∼107 mAh/g after 150 cycles (∼86% capacity retention), indicating the best cycling stability. The Ca 0.1 LNMO also shows enhanced stability relative to the undoped sample, retaining ∼102 mAh/g after 150 cycles (∼84% retention), whereas Ca 0.01 LNMO exhibits intermediate behavior (∼91 mAh/g after 150 cycles, ∼79% retention). The improved cycling stability indicates that Ca doping mitigates both bulk structural degradation and interfacial parasitic reactions during high-voltage cycling. From a bulk perspective, partial substitution by Ca^2+^ reduces the fraction of Mn^3+^, thereby suppressing Jahn–Teller distortion and lattice instability, which are known to accelerate transition-metal dissolution in spinel LNMO.^4^ In addition, the slightly expanded lattice parameter (Figure 3d) and reduced polarization (Figure 7d) suggest enhanced structural robustness during Li^+^ insertion/extraction. From an interfacial perspective, the smaller impedance growth observed in electrochemical impedance spectroscopy (Figure 7b) indicates reduced charge-transfer resistance (R_CT_) and interphase resistance (R_CEI_), suggesting slower CEI growth and reduced electrolyte decomposition. While direct postmortem surface chemical analysis was not conducted in this study, the combination of improved capacity retention, lower polarization growth, and reduced impedance evolution consistently supports mitigated transition-metal dissolution and interfacial degradation.

The voltage profiles and corresponding dQ/dV profiles of LNMO at certain cycles further elucidate the underlying mechanism of the observed cycling behavior (Figure 8). For undoped LNMO, a strong and evolving peak at ∼3.8–4.2 V corresponds to the presence of Mn^3+^/Mn^4+^ redox, which broadens and shifts with cycling (Figure 8a and b), reflecting increasing polarization and side reactions associated with oxygen-vacancy-induced Mn dissolution. In contrast, the Ca 0.05 sample exhibits a markedly attenuated 4.0 V peak whose intensity and position remain nearly invariant over prolonged cycling (Figure 8c and d), indicating the suppression of Mn^3+^ formation and stabilization of the interphase. This stability correlates directly with its superior retention of ∼94% after 500 cycles. Notably, the Ca 0.1 sample shows the most stable ∼4 V peak through 500 cycles (Figure 8e and f). However, its capacity retention is lower compared to Ca 0.05. This paradox arises because higher Ca causes bulk distortions (Ni/Mn ordering and lattice strain) that increase transport resistance even with stabilized Mn^3+^ chemistry. In contrast, Ca 0.05 achieves the optimal balance. It suppresses Mn^3+^-driven side reactions and stabilizes the ∼4 V plateau, while maintaining a disordered *Fd*3̅*m* framework that promotes a solid-solution reaction rather than a two-phase transition, thereby mitigating voltage polarization during cycling.^1,30^ Overall, moderate doping (Ca 0.05) optimally combines interfacial stability with fast Li^+^ transport, whereas excessive doping (Ca 0.1) introduces partial ordering, leading to reduced electrochemical performance.

**Figure 8:**
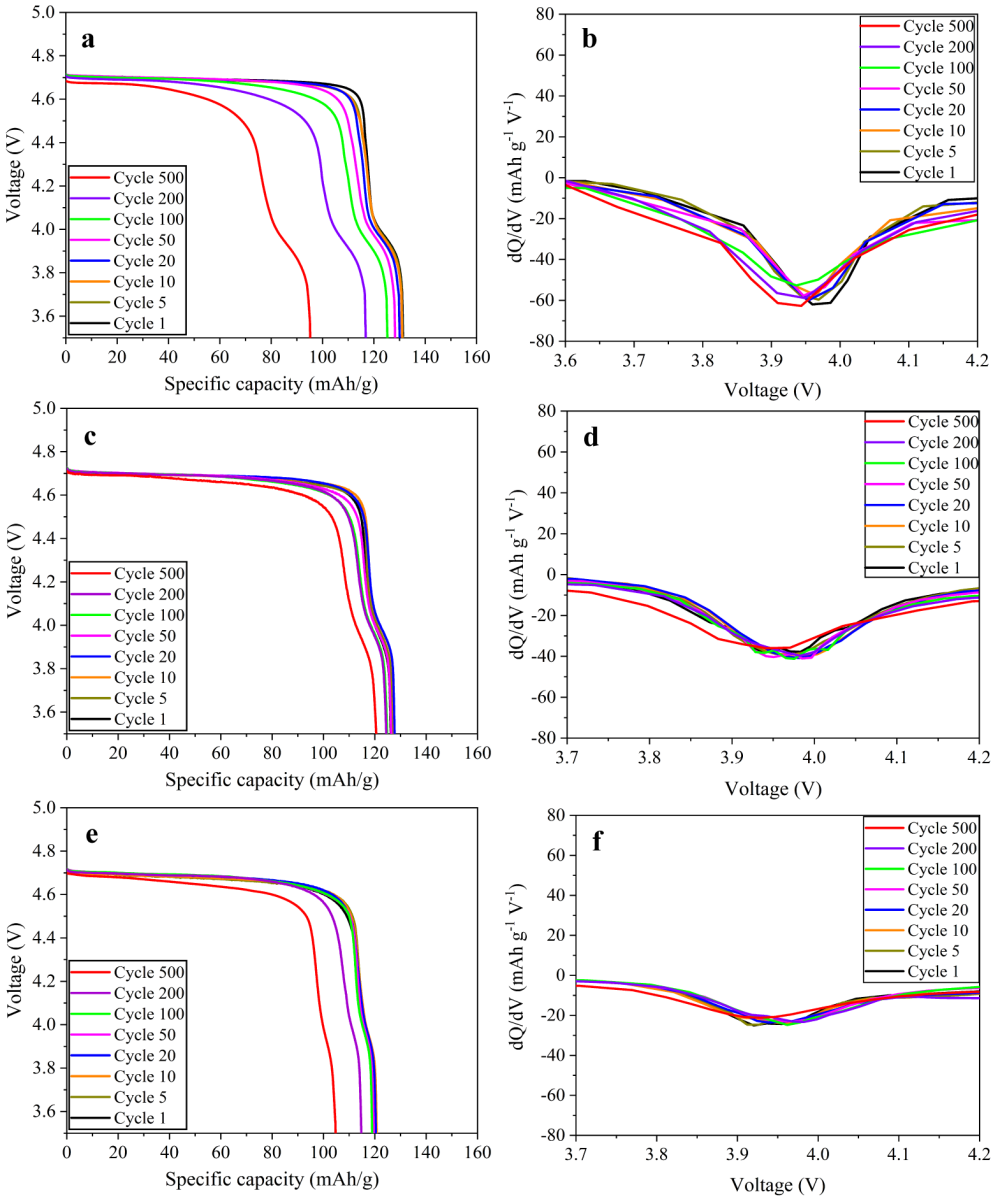
Voltage profiles (a, c, and e) and corresponding dQ/dV plots (b, d, and f) of undoped (a, b), Ca 0.05 (c, d), and Ca 0.1 (e, f) LNMO cathodes at various cycles.

## 4 Conclusions

In this study, we successfully identified a cost-effective element with a strong bonding effect to oxygen and a large ionic radius as an effective dopant to improve the electrochemical performance of LNMO cathodes. The preliminary screening results showed that among the three dopants Ca, Sr, and Cr studied here, the moderate ionic radius and strong affinity to oxygen enabled Ca to be the most suitable dopant for the LNMO cathode exhibiting the highest initial discharge capacity (∼124 mAh/g) and cycling stability (∼94% retention after 500 cycles).

We have also successfully revealed the dependence of the crystal, morphological, and electrochemical properties of the LNMO cathode on the Ca doping level and identified Ca 0.05 as the optimal doping level for LNMO cathode materials in terms of the electrochemical performance of derived cells. Results from XRD, SEM, and TEM confirmed that Ca is successfully incorporated into the spinel lattice. At moderate Ca doping (Ca 0.05), the obtained LNMO cathodes have a disordered *Fd*3̅*m* framework, expanded lattice parameters, suppressed high-energy {100} facets, and promoted well-faceted octahedra showing a high ratio of {111} planes. However, excessive Ca doping (e.g., Ca 0.1) induced partial Ni/Mn ordering in LNMO. Electrochemical measurements also demonstrated that Ca 0.05 LNMO showed the lowest Mn^3+^ redox contribution, reduced polarization, and superior kinetic properties. These structural and kinetic advantages enabled the Ca 0.05 cathode to retain a specific capacity of ∼121 mAh/g (∼94.4%) after 500 cycles at 0.5 C and deliver ∼111 mAh/g at 10 C. By contrast, undoped LNMO exhibited rapid capacity fading and strong polarization growth, while Ca 0.1 showed reduced rate and cycling stability due to ordering-related transport penalties. Combined with results from material characterizations, the electrochemical analysis clearly elucidates the structure–property–performance relationships of LNMO cathode materials.

The Ca-doped LNMO cathode materials developed here have the advantages of low cost, high rate capability, and long cycle life and can be utilized as cathode materials in practical LIBs directly. The method used here to dope large elements into cathode materials through the coprecipitation route is universal and can be easily adopted and extended to integrate other dopants into cathode materials for targeted properties.

## Supplementary Material



## Notes

Additional EDS mappings, EPMA line profiles, Rietveld refinement profiles of XRD patterns, electrochemical data, and performance comparison tables (PDF)

## References

[1] Liang G., Peterson V. K., See K. W., Guo Z., Pang W. K. (2020). Developing
high-voltage spinel LiNi 0.5 Mn 1.5 O 4 cathodes for high-energy-density
lithium-ion batteries: current achievements and future prospects. J. Mater. Chem. A.

[2] Pieczonka N. P., Liu Z., Lu P., Olson K. L., Moote J., Powell B. R., Kim J.-H. (2013). Understanding
transition-metal dissolution behavior
in LiNi0. 5Mn1. 5O4 high-voltage spinel for lithium ion batteries. J. Phys. Chem. C.

[3] Kim J.-H., Pieczonka N. P., Li Z., Wu Y., Harris S., Powell B. R. (2013). Understanding the
capacity fading mechanism in LiNi0.
5Mn1. 5O4/graphite Li-ion batteries. Electrochim.
Acta.

[4] Lu D., Yuan L., Li J., Huang R., Guo J., Cai Y. (2015). Failure mechanism for
high voltage graphite/LiNi0. 5Mn1. 5O4 (LNMO)
Li-ion cells stored at elevated temperature. J. Electroanal. Chem..

[5] Deng H., Nie P., Luo H., Zhang Y., Wang J., Zhang X. (2014). Highly enhanced
lithium storage capability of LiNi 0.5 Mn 1.5 O 4 by coating with
Li 2 TiO 3 for Li-ion batteries. J. Mater. Chem.
A.

[6] Mereacre V., Stüble P., Trouillet V., Ahmed S., Volz K., Binder J. R. (2023). Coating versus Doping: Understanding the Enhanced Performance
of High-Voltage Batteries by the Coating of Spinel LiNi0. 5Mn1. 5O4
with Li0. 35La0. 55TiO3. Adv. Mater. Interfaces.

[7] Park N. R., Li Y., Yao W., Zhang M., Han B., Mejia C., Sayahpour B., Shimizu R., Bhamwala B., Dang B. (2024). Understanding the role of lithium borate as the surface
coating on
high voltage single crystal LiNi0. 5Mn1. 5O4. Adv. Funct. Mater..

[8] Fang X., Ge M., Rong J., Che Y., Aroonyadet N., Wang X., Liu Y., Zhang A., Zhou C. (2014). Ultrathin
surface modification by atomic layer deposition on high voltage cathode
LiNi0. 5Mn1. 5O4 for lithium ion batteries. Energy Technol..

[9] Kim H., Byun D., Chang W., Jung H.-G., Choi W. (2017). A nano-LiNbO
3 coating layer and diffusion-induced surface control towards high-performance
5 V spinel cathodes for rechargeable batteries. J. Mater. Chem. A.

[10] Chang L.-J., Cao S.-Y., Luo S.-H., Li K., Bi X.-L., Wei A.-L., Liu J.-N. (2021). Effect of Different
Calcination Temperatures
on the High-Temperature Properties of AlPO4-Coated Modified Spinel
LiNi0. 5Mn1. 5O4 Material. Energy Technol..

[11] Liang G., Wu Z., Didier C., Zhang W., Cuan J., Li B., Ko K. Y., Hung P. Y., Lu C. Z., Chen Y. (2020). A long cycle-life high-voltage
spinel lithium-ion battery electrode
achieved by site-selective doping. Angew. Chem.,
Int. Ed..

[12] Stüble P., Geßwein H., Indris S., Müller M., Binder J. R. (2022). On the electrochemical properties of the Fe–Ti
doped LNMO material LiNi 0.5 Mn 1.37 Fe 0.1 Ti 0.03 O 3.95. J. Mater. Chem. A.

[13] Chen A., Kong L., Shu Y., Yan W., Wu W., Xu Y., Gao H., Jin Y. (2019). Role of Al-doping
with different
sites upon the structure and electrochemical performance of spherical
LiNi 0.5 Mn 1.5 O 4 cathode materials for lithium-ion batteries. RSC Adv..

[14] Mao J., Dai K., Xuan M., Shao G., Qiao R., Yang W., Battaglia V. S., Liu G. (2016). Effect of chromium and niobium doping
on the morphology and electrochemical performance of high-voltage
spinel LiNi0. 5Mn1. 5O4 cathode material. ACS
Appl. Mater. Interfaces.

[15] Chladil L., Kunický D., Kazda T., Vanýsek P., Čech O., Bača P. (2021). In-situ XRD study of a Chromium doped
LiNi0. 5Mn1. 5O4 cathode for Li-ion battery. J. Energy Storage.

[16] Wang Y.-Y., Song X., Liu S., Li G.-R., Ye S.-H., Gao X.-P. (2021). Elucidating the effect of the dopant ionic radius on
the structure and electrochemical performance of Ni-rich layered oxides
for lithium-ion batteries. ACS Appl. Mater.
Interfaces.

[17] Wei A., Mu J., He R., Bai X., Li X., Zhang L., Wang Y., Liu Z., Wang S. (2021). Enhanced electrochemical
performance of LiNi0. 5Mn1. 5O4 composite cathodes for lithium-ion
batteries by selective doping of K+/Cl– and K+/F–. Nanomaterials.

[18] Haynes, W. M. . CRC handbook of chemistry and physics; CRC press, 2016.

[19] Wang J., Wang Y., Zheng M., Cai F. (2025). Strontium Doping Promotes
Low-Temperature Growth of Single-Crystalline Ni-Rich Cathodes with
Enhanced Electrochemical Performance. Materials.

[20] Luo Y., Cui Z., Wu C., Sa B., Wen C., Li H., Huang J., Xu C., Xu Z. (2023). Enhanced electrochemical
performance of a Ti–Cr-Doped LiMn1. 5Ni0. 5O4 cathode material
for lithium-ion batteries. ACS Omega.

[21] Dang Y., Phuah X. L., Wang H., Yang B., Wang H., West A. R. (2021). Electrical properties and charge compensation mechanisms
of Cr-doped rutile, TiO 2. Phys. Chem. Chem.
Phys..

[22] Liu G., Zhang J., Zhang X., Du Y., Zhang K., Li G., Yu H., Li C., Li Z., Sun Q. (2017). Study on oxygen deficiency in spinel LiNi0. 5Mn1. 5O4 and its Fe
and Cr-doped compounds. J. Alloys Compd..

[23] Aktekin B., Massel F., Ahmadi M., Valvo M., Hahlin M., Zipprich W., Marzano F., Duda L., Younesi R., Edström K. (2020). How Mn/Ni ordering controls electrochemical
performance in high-voltage spinel LiNi0. 44Mn1. 56O4 with fixed oxygen
content. ACS Appl. Energy Mater..

[24] Shannon R. D. (1976). Revised
effective ionic radii and systematic studies of interatomic distances
in halides and chalcogenides. Found. Crystallogr..

[25] Lin Y., Välikangas J., Sliz R., Molaiyan P., Hu T., Lassi U. (2023). Optimized
morphology and tuning the Mn3+ content of LiNi0. 5Mn1.
5O4 cathode material for Li-ion batteries. Materials.

[26] Li Z.-Q., Liu Y.-F., Liu H.-X., Zhu Y.-F., Wang J., Zhang M., Qiu L., Guo X.-D., Chou S.-L., Xiao Y. (2024). Kinetically controlled
synthesis of low-strain disordered micro–nano
high voltage spinel cathodes with exposed {111} facets. Chem. Sci..

[27] Wan L., Deng Y., Yang C., Xu H., Qin X., Chen G. (2015). Ni/Mn ratio and morphology-dependent
crystallographic facet structure
and electrochemical properties of the high-voltage spinel LiNi 0.5
Mn 1.5 O 4 cathode material. RSC Adv..

[28] Liu Z., Wen X., Wu X., Gao Y., Chen H., Zhu J., Chu P. (2009). Intrinsic dipole-field-driven
mesoscale crystallization of core–
shell ZnO mesocrystal microspheres. J. Am. Chem.
Soc..

[29] Wang L., He X., Sun W., Wang J., Li Y., Fan S. (2012). Crystal orientation
tuning of LiFePO4 nanoplates for high rate lithium battery cathode
materials. Nano Lett..

[30] Xiao J., Chen X., Sushko P. V., Sushko M. L., Kovarik L., Feng J., Deng Z., Zheng J., Graff G. L., Nie Z. (2012). High-performance
LiNi0. 5Mn1. 5O4 spinel controlled
by Mn3+ concentration and site disorder. Adv.
Mater..

[31] Ding H., Wang P., Zhang N., Zhou J., Li X., Li C., Zhao D., Li S. (2024). Improving electrochemical performances
of LiNi0. 5Mn1. 5O4 by the strategy of oxygen vacancy doping. Appl. Mater. Today.

[32] James, G. S. Lange’s Handbook of Chemistry; McGRAW-HILL, INC., 2005.

[33] Gajraj V., Azmi R., Darma M., Indris S., Ehrenberg H., Mariappan C. (2021). Correlation
between structural, electrical and electrochemical
performance of Zn doped high voltage spinel LiNi0. 5-xZnxMn1. 5O4
porous microspheres as a cathode material for Li-Ion batteries. Ceram. Int..

[34] Rahim A. S. A., Kufian M. Z., Arof A. K. M., Osman Z. (2022). Variation of Li Diffusion
Coefficient during Delithiation of Spinel LiNi 0.5 Mn 1.5 O 4. J. Electrochem. Sci. Technol..

[35] Chien Y.-C., Liu H., Menon A. S., Brant W. R., Brandell D., Lacey M. J. (2023). Rapid determination
of solid-state diffusion coefficients in Li-based batteries via intermittent
current interruption method. Nat. Commun..

[36] Jin J., Wei J., Zhou Z., Xie Z. (2023). Application of 5V spinel material
LiNi 0.5 Mn 1.5 O 4 in Li-ion batteries: single crystalline or polycrystalline?. RSC Adv..

