# Supplementary material for: Calcium-Doped High-Voltage Spinel Cathode for Long Cycle Life Lithium-Ion Batteries
Source: ACS Appl Energy Mater. 2026 Mar 17;9(7):4016–29. doi: 10.1021/acsaem.6c00023 (PMC13080766; doi:10.1021/acsaem.6c00023)
Supplement: Supplementary file 1 [file ae6c00023_si_001.pdf]

(Supporting information)

Calcium Doped High-Voltage Spinel Cathode for Long Cycle Life Lithium-Ion Batteries

Jie Xiong[a], Bingyao Zhou[a], Kevin Mathew[a], Emmanuel Kornyo[a], Guoxin Zhang[a], Wenquan Lu[b], Zhi Mei[c], Qingliu Wu[a,]*

a- Department of Chemical and Paper Engineering, Western Michigan University, 4601 Campus Drive, Kalamazoo, Michigan, 49008-5462, U. S. A.

b- Chemical Science and Engineering Division, Argonne National Laboratory, 9700 South Cass Ave., Lemont, Illinois, 60439-4837, U. S. A.

c- Lumigen Instrument Center, Wayne State University, 5101 Cass Ave, Detroit, Michigan, 48202, U. S. A.

* Correspondence should be addressed to:

Qingliu Wu, email: qingliu.wu@wmich.edu; Phone: 269-276-3998; Fax: 269-276-3501

Figure S1. Specific capacity (a) and capacity retention (b) of Ca, Sr, and Cr doped LNMO cathodes as a function of cycle numbers at 25 ℃ and 0.5C.

Figure S2. Cross-section image (a) and EPMA line profiles for Ni, Mn and Ca along the marked white line in cross-section image of Ca 0.05 LNMO particle. Zoomed view (c) showing a uniform bulk distribution without surface segregation. The scale bar represents 1 μm.

Figure S3. Rietveld refinement profiles of XRD data of undoped (a), Ca 0.01 (b), Ca 0.05 (c), and Ca 0.1 (d) LNMO samples. The black dots represent the experimental data and the solid blue lines are for Rietveld refinement fit. The lower red curves are the difference between the observed and calculated at each step.

Figure S4. Elemental mappings of undoped (a), Ca 0.05 (b) and Ca 0.1 (c) LNMO cathode samples.

Figure S5. Specific capacity of Ca doped LNMO cathodes at various current densities as a function of cycle number.

Figure S6. Voltage profiles of undoped and Ca 0.05 LNMO cathodes measured at 0.2C, 2C, and 10C (third cycle at each C-rate) at 25 ℃.

Figure S7. Li^+ diffusion coefficients (D_{Li^+}) for undoped and Ca 0.05 LNMO measured by galvanostatic intermittent titration technique (GITT) during discharge.

Table S1. Ionic radius and M-O bond dissociation energies of commonly used cation dopants for LIB cathodes.

Table S2. ICP-OES results of undoped and Ca 0.05 LNMO samples.

Table S3. Rietveld refinement results of Ca-doped LNMO cathodes.

Table S4. Summary of peak intensity ratios calculated from XRD patterns collected from Ca-doped LNMO samples.

Table S5. Discharge capacity contribution from Mn^{3+} plateau (3.8 – 4.2 V) in the third formation cycle on Ca-doped LNMO cathodes.

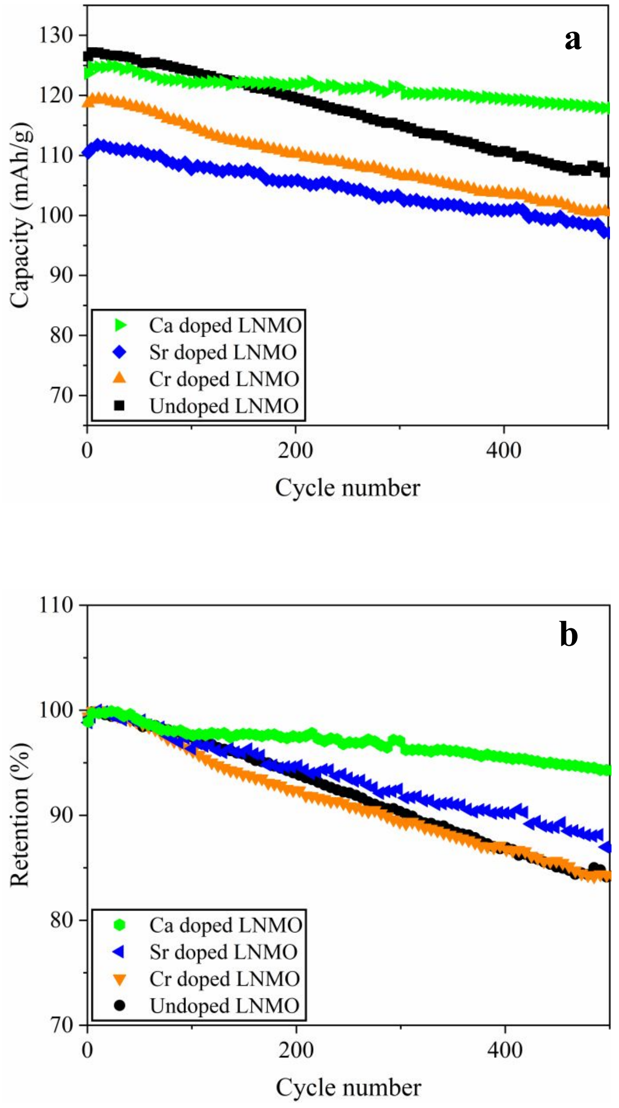

Figure S1. Specific capacity (a) and capacity retention (b) of Ca, Sr, and Cr doped LNMO cathodes as a function of cycle numbers at 25 °C and 0.5C.

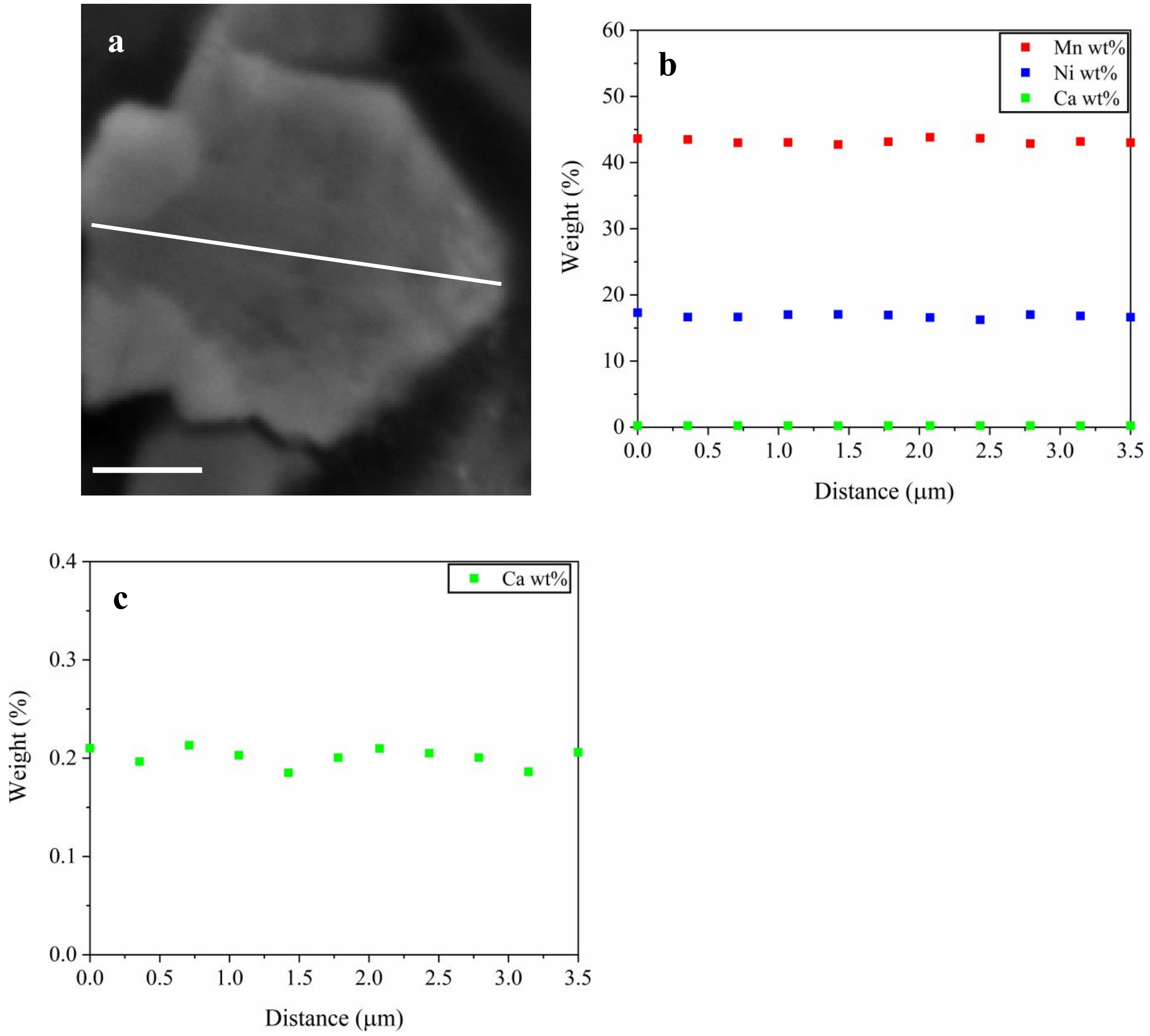
a
b
Mn wt%
Ni wt%
Ca wt%
Weight (%)
Distance (μm)
0
10
20
30
40
50
60
0.0
0.5
1.0
1.5
2.0
2.5
3.0
3.5
c
Ca wt%
Weight (%)
Distance (μm)
0.0
0.1
0.2
0.3
0.4
0.0
0.5
1.0
1.5
2.0
2.5
3.0
3.5

Figure S2. Cross-section image (a) and EPMA line profiles for Ni, Mn and Ca along the marked white line in cross-section image of Ca 0.05 LNMO particle. Zoomed view (c) showing a uniform bulk distribution without surface segregation. The scale bar represents 1 μm.

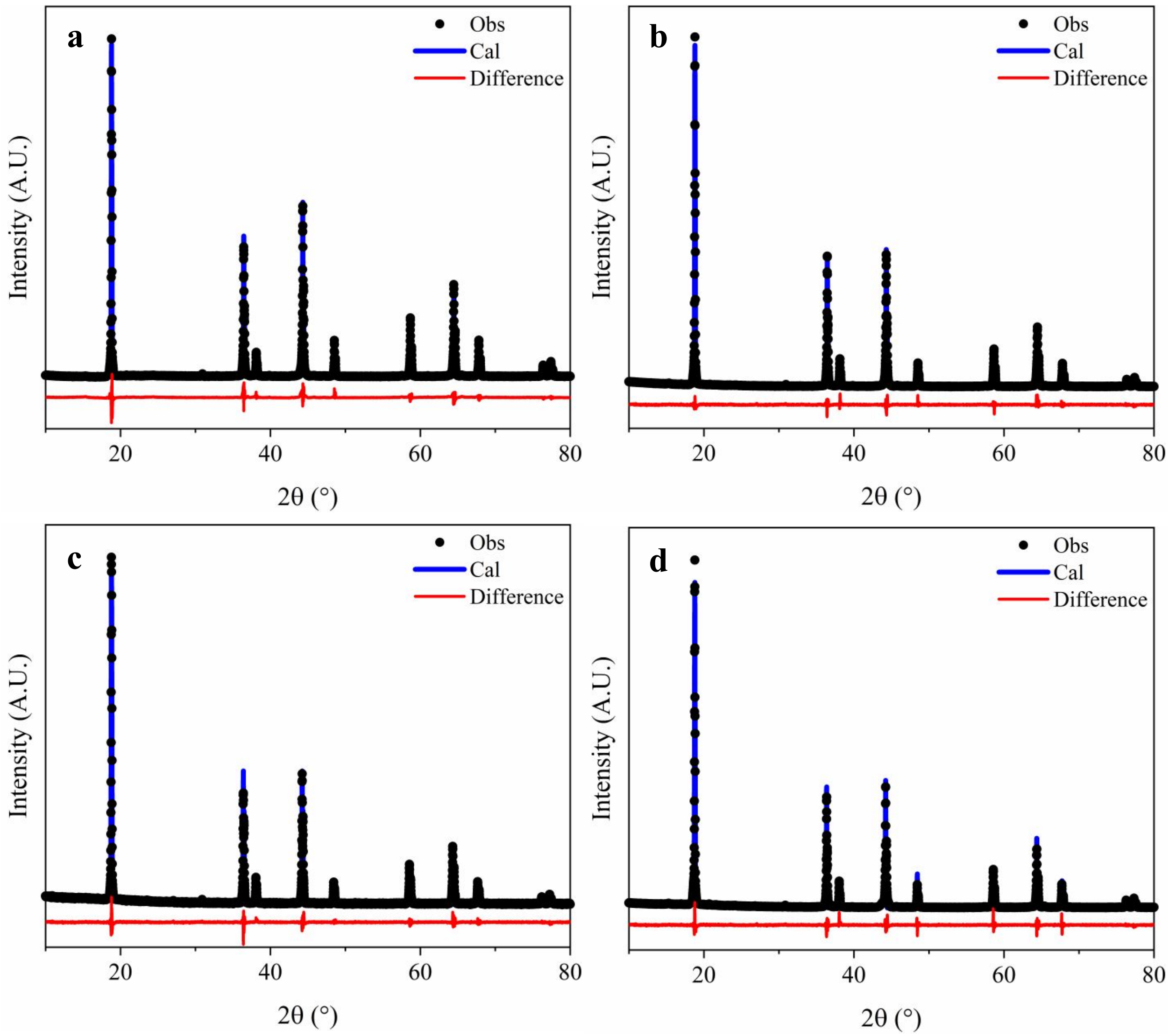

Figure S3. Rietveld refinement profiles of XRD data of undoped (a), Ca 0.01 (b), Ca 0.05 (c), and Ca 0.1 (d) LNMO samples. The black dots represent the experimental data and the solid blue lines are for Rietveld refinement fit. The lower red curves are the difference between the observed and calculated at each step.

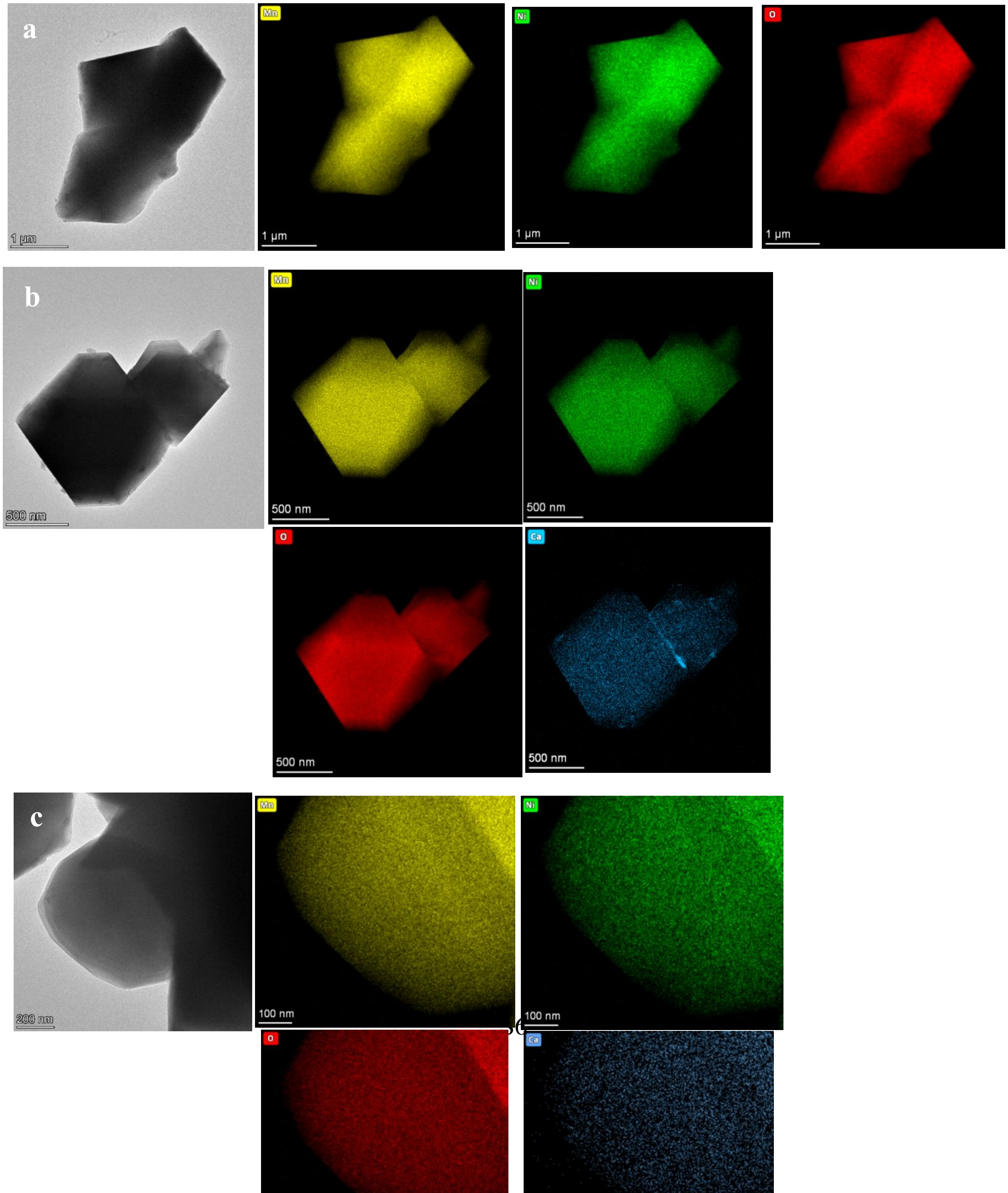

Figure S4. Elemental mappings of undoped (a), Ca 0.05 (b) and Ca 0.1 (c) LNMO cathode samples.

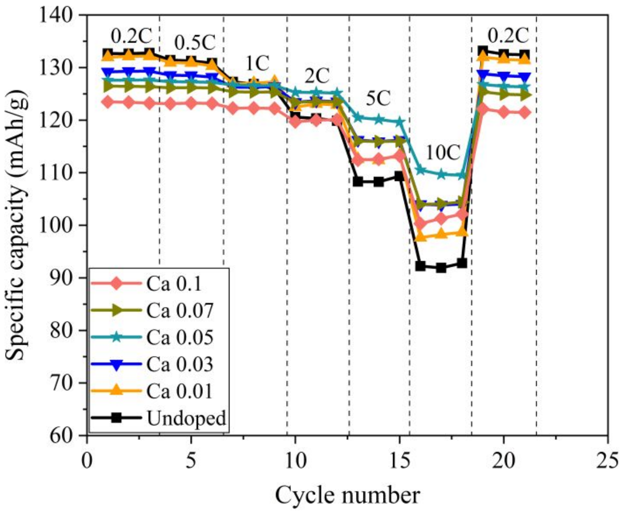

Figure S5. Specific capacity of Ca doped LNMO cathodes at various current densities as a function of cycle number.

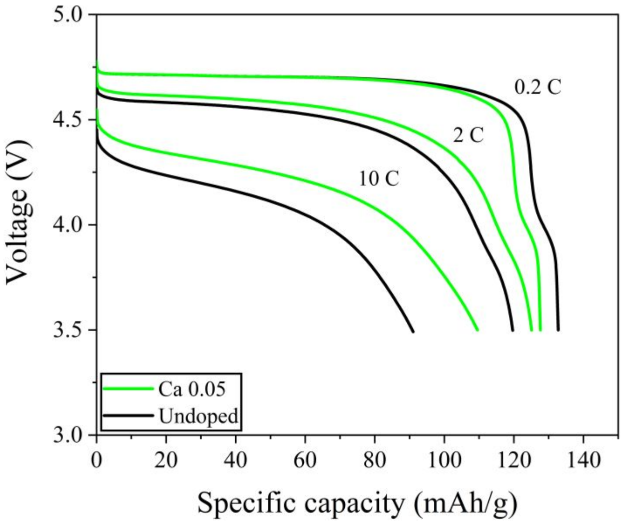

Figure S6. Voltage profiles of undoped and Ca 0.05 LNMO cathodes measured at 0.2C, 2C, and 10C (third cycle at each C-rate) at 25 °C.

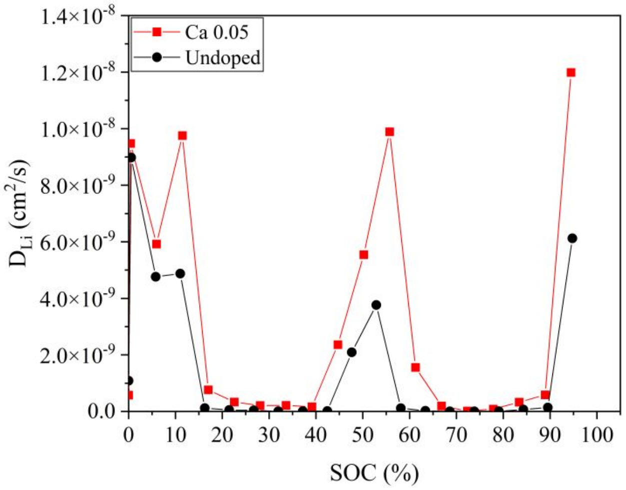

Figure S7. Li^+ diffusion coefficients (D_{Li^+}) for undoped and Ca 0.05 LNMO measured by galvanostatic intermittent titration technique (GITT) during discharge.

Table S1. Ionic radius and M-O bond dissociation energies of commonly used cation dopants for LIB cathodes.

Dopant	Ionic radius (Å, CN=6)	M-O bonding strength (kJ/mol)	Ionic potential ($Å^{-1}$)
Ca	1.00	~ 464	2.00
Sr	1.18	~ 454	1.70
Cr	0.62	~ 427	4.84
Mg	0.72	~ 418	2.78
Fe	0.65	~ 407	4.62
Zn	0.74	~ 290	2.70

Table S2. ICP-OES results of undoped and Ca 0.05 LNMO samples.

	Li	Ca	Mn	Ni
Baseline	1.01	0	1.51	0.49
Ca 0.05	1.00	0.04	1.47	0.49

Table S3. Rietveld refinement results of Ca-doped LNMO cathodes.

Sample	a (Å)	V (Å^3)	Rp (%)	R_{wp} (%)	R_{exp} (%)	Reduced χ^2	GOF
Undoped	8.168	544.94	2.38	4.09	1.85	4.89	2.21
Ca 0.01	8.171	545.54	2.46	3.85	1.80	4.57	2.14
Ca 0.05	8.182	547.75	2.51	3.62	1.77	4.18	2.05
Ca 0.1	8.194	550.16	2.58	3.7	1.78	4.32	2.08

Table S4. Summary of peak intensity ratios calculated from XRD patterns collected from Ca-doped LNMO samples.

Sample	I(111)/I(311)	I(311)/I(400)	I(400)/I(111)
Undoped	1.94	1.04	0.49
Ca 0.01	2.68	1.10	0.34
Ca 0.03	2.92	1.10	0.31
Ca 0.05	3.12	1.09	0.29
Ca 0.07	3.13	1.03	0.31
Ca 0.1	3.14	0.96	0.33

Table S5. Discharge capacity contribution from Mn^{3+} plateau (3.8 – 4.2 V) in the third formation cycle on Ca-doped LNMO cathodes.

Sample	3.8 V – 4.2 V capacity (mAh/g)	Total discharge capacity (mAh/g)	3.8 V – 4.2 capacity ratio (%)
Undoped	12.11	131.40	9.21
Ca 0.01	9.96	130.32	7.64
Ca 0.03	8.21	128.60	6.39
Ca 0.05	7.65	126.26	6.06
Ca 0.07	6.59	124.36	5.30
Ca 0.1	5.72	120.52	4.75
